# Natural Killer Cell Membrane‐Cloaked Virus‐Mimicking Nanogenerator with NIR‐Triggered Shape Reversal and •C/•OH Storm for Synergistic Thermodynamic–Chemodynamic Therapy

**DOI:** 10.1002/advs.202103498

**Published:** 2021-12-19

**Authors:** Jinyan Lin, Yang Li, Peiyuan Wang, Ming Wu, Fukai Zhu, Yun Zhang, Zhenqing Hou, Jingfeng Liu, Xiaolong Liu

**Affiliations:** ^1^ The United Innovation of Mengchao Hepatobiliary Technology Key Laboratory of Fujian Province Mengchao Hepatobiliary Hospital of Fujian Medical University Fuzhou 350025 P. R. China; ^2^ CAS Key Laboratory of Design and Assembly of Functional Nanostructures, Fujian Institute of Research on the Structure of Matter Chinese Academy of Sciences Fuzhou 350002 P. R. China; ^3^ Department of Translational Medicine, Xiamen Institute of Rare Earth Materials Chinese Academy of Sciences Xiamen 361024 P. R. China; ^4^ College of Materials Xiamen University Xiamen 361005 P. R. China; ^5^ Fujian Cancer Hospital and Fujian Medical University Cancer Hospital Fuzhou 350014 P. R. China

**Keywords:** immune escape, NIR‐triggered shape reversal, natural killer cell membrane, thermodynamic–chemodynamic therapy, virus‐mimicking nanogenerators

## Abstract

Free radical‐based anticancer modality has been widely applied to cancer therapies. However, it still faces challenges of low delivery efficiency and poor selectivity of free radical generation specifically toward tumors. Herein, a virus‐mimicking hollow mesoporous disulfide‐bridged organosilica is designed to encapsulate •C precursor 2, 2'‐azobis[2‐(2‐imidazolin‐2‐yl) propane] dihydrochloride (AIPH), which is then enclosed by tannic acid (TA)/Fe^III^ photothermal assembly and further cloaked by natural killer (NK) cell membrane to achieve synergistic thermodynamic–chemodynamic therapy. The nanogenerator can first evade immune surveillance via NK cell membrane “cloaking” mechanism to strongly accumulate in tumors. Interestingly, the NIR laser‐induced heat can trigger NK cell membrane rupture for “shape reversal” to expose a virus‐like surface to amplify the cellular uptake, and simultaneously break the azo bonds of AIPH for in situ controlled •C generation. Then upon glutathione (GSH) triggering, the nanogenerator disintegrates via disulfide–thiol exchange and efficiently generates •OH by lysosomal pH‐initiated TA‐Fe^III^ reaction; notably, the consumption of GSH can amplify oxidative stress to enhance free radical therapy by weakening the self‐defense mechanism of tumor cells. It is envisioned that the NK cell membrane‐cloaked virus‐mimicking and NIR/GSH sequentially activated •C/•OH radical nanogenerator can provide a promising strategy for oxidative stress‐based anticancer therapy.

## Graphical abstract

A strategy of “biomimetic engineering‐mediated cloaking followed by NIR‐triggered shape reversal” is proposed to amplify the synergistic thermodynamic–chemodynamic therapeutic effect via •C/•OH storm.

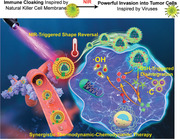

## 1 Introduction

Oxidative stress is a phenomenon of excessive free radicals or other reactive oxygen species (ROS) accumulation in cells.^[^ ^1^, ^2^ ^]^ Free radicals are highly reactive molecules, which can easily bind to surrounding substances (including lipids, proteins, and nucleic acids) and trigger cell death by the oxidation reaction.^[^ ^3^, ^4^ ^]^ Compared with normal cells, cancer cells undergo severer oxidative stress, indicating that they are more vulnerable to oxidative species.^[^ ^5^ ^]^ Based on this, radiotherapy and a variety of chemotherapy drugs are developed to inhibit tumors by producing superfluous free radicals.^[^ ^6^, ^7^, ^8^ ^]^ Recently, antitumor strategies on the strength of free radicals have been extensively applied to chemodynamic therapy^[^ ^9^ ^]^ and thermodynamic therapy.^[^ ^10^, ^11^ ^]^ Chemodynamic therapy is an emerging anticancer modality, which catalyzes less‐reactive hydrogen peroxide (H_2_O_2_) into highly oxidative hydroxyl radical (•OH) through Fenton/Fenton‐like reaction to induce oxidative stress in tumor cells.^[^ ^12^ ^]^ Compared with traditional chemotherapy and radiotherapy, chemodynamic therapy is activated by endogenous overproduced H_2_O_2_ of cancer cells, endowing it with high selectivity toward tumor tissues rather than normal tissues. However, the chemodynamic therapeutic efficiency largely depends on the pH value (the optical pH is 3–4),^[^ ^13^ ^]^ which is difficult to achieve in tumor microenvironment, resulting in insufficient production of free radicals to get satisfactory anticancer outcomes. Thus, it is essential to explore other available free radicals to destroy tumor more efficiently.^[^ ^14^, ^15^ ^]^ Very recently, a feasible alternative to produce alkyl radical (•C) has been proposed for thermodynamic therapy of cancer, in which one kind of azoamidine compounds 2,2‐azobis[2‐(2‐imidazolin‐2‐yl) propane] dihydrochloride (AIPH) was utilized as the radical precursor.^[^ ^16^ ^]^ Under thermal triggering, AIPH can be rapidly decomposed to produce toxic alkyl radical (•C) to kill cancer cells by directly oxidizing cellular substances or augmenting intracellular lipid hydroperoxides.^[^ ^17^, ^18^ ^]^ However, the AIPH is thermally unstable and the free radical production efficiency is highly dependent on the temperature in biological environment, it urgently needs to engineer a heat producer to control •C generation at tumor sites.

To realize above assumption, photothermal therapy could be utilized to generate hyperthermia to increase the yield of •C from AIPH. Simultaneously, it is an effective method to improve the chemodynamic therapeutic efficiency since the elevation of temperature could accelerate Fenton/Fenton‐like reaction rate.^[^ ^19^ ^]^ Up to now, new types of photothermal agents, such as gold nanocages,^[^ ^14^ ^]^ magnetic Fe_5_C_2_ nanoparticles,^[^ ^20^ ^]^ CuFeSe_2_ nanoparticles,^[^ ^21^ ^]^ and Nb_2_C Mxene nanosheets,^[^ ^11^ ^]^ have been developed as the in situ heat sources to achieve photothermal‐augmented free radical‐based therapy (chemodynamic therapy, sonodynamic therapy, thermodynamic therapy, etc.). Nevertheless, the therapeutic effect of this anticancer modality is easily attenuated by free radical scavenging of intracellular antioxidants.^[^ ^22^, ^23^ ^]^ As one of the primary endogenous antioxidants, glutathione (GSH) is over‐produced and directly participates in the elimination of free radicals in tumor cells to maintain redox homeostasis, thereby attenuating the therapeutic efficiency. Therefore, designing a nanogenerator with the ability of free radical generation and intracellular GSH consumption could simultaneously amplify oxidative stress to achieve more effective anticancer therapy. However, free radicals are likewise noxious to normal cells and their therapeutic effect would be significantly impeded due to the insufficient enrichment of nanogenerators both within the tumor tissues and inside the tumor cells. Thus, it is very crucial to improve the tumor accumulation/tumor cell entry of nanogenerators and spatiotemporally control free radical generation and release within tumor cells to improve therapeutic effects while reducing side effects.

The manipulation of micro‐topography such as geometrical shape and surface topography of nanoparticles could provide a perspective to promote their cell entry.^[^ ^24^, ^25^, ^26^, ^27^, ^28^ ^]^ Lately, some studies showed that the virus‐mimicking nanoparticles could be uptaken by cells more efficiently compared with conventional sphere‐shaped ones.^[^ ^14^, ^27^, ^29^ ^]^ Nevertheless, directly exposing virus‐mimicking micro‐topography on the nanogenerators’ surface would inevitably suffer serious drawbacks of rapid clearance in the blood circulation due to immune surveillance, which would significantly weaken the delivery efficiency of nanogenerators to tumor regions. To overcome this conflict, a protection–deprotection strategy based on tumor microenvironment or external stimulus via introducing sheddable shielding blocks onto the nanomaterials’ surface could be undoubtedly adopted.^[^ ^30^, ^31^ ^]^ Recently, the novel variety of cytomembrane‐camouflaged nanosystems have attracted increasing attentions.^[^ ^32^, ^33^ ^]^ It has been widely reported that the erythrocyte and macrophagocyte membrane‐coated nanoparticles exhibited the unique ability of prolonging circulation time and reducing macrophage uptake, respectively.^[^ ^34^, ^35^ ^]^ This promising strategy inspires us to develop natural killer (NK) cell membrane‐cloaked nanoparticles for cancer therapy. NK cells are lymphocytes of the innate immune system that play a vital role in tumor immunosurveillance and can accumulate into the tumor tissues.^[^ ^36^, ^37^ ^]^ Born to kill, the NK cells were derived from bone marrow, circulating throughout the body, to identify tumor cells. Thus, it can be expected that introducing NK cell membrane onto the virus‐mimicking nanoparticles can evade immune surveillance and enhance the tumor accumulation. Additionally, once irradiated by external NIR laser, the NK cell membrane could be ruptured by “thermal disturbance”^[^ ^38^ ^]^ to expose the virus‐mimicking surface topography, which would promote the intracellular uptake.

**Scheme 1:**
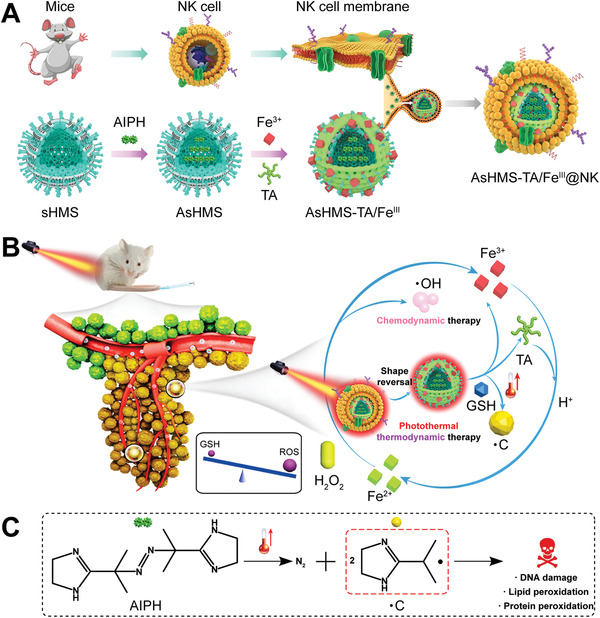
A) Schematic illustration of the synthesis of NK cell membrane‐cloaked virus‐mimicking AsHMS‐TA/Fe^III^@NK nanogenerator. B) Schematic illustration of AsHMS‐TA/Fe^III^@NK nanogenerator for NIR/GSH‐triggered shape reversal and •C/•OH release for photothermal‐primed thermodynamic–hemodynamic therapy. C) Schematic illustration of thermal decomposition of AIPH.

In view of above considerations, herein, the NK cell membrane‐cloaked virus‐mimicking and NIR/GSH sequentially activated free radical nanogenerator was developed to efficiently invade tumor cells and initiate controllable release of •C/•OH storm for synergistic thermodynamic–chemodynamic therapy (**Scheme** **1**). The nanogenerator was consisted of outmost NK cell membrane shell and virus‐like disulfide‐doped hollow mesoporous silica (sHMS) core which was loaded with thermal azo‐initiator (AIPH) and decorated with tannic acid (TA)/Fe^3+^ coordination (designed as AsHMS‐TA/Fe^III^@NK). First, the NK cell membrane could protect nanogenerator from immune elimination to achieve high tumor accumulation. Once irradiated by the NIR laser, it would be ruptured to realize the “sphere‐to‐virus” shape transition of nanogenerator, thereby promoting tumor cell invasion via spike surface‐assisted adhesion. The TA/Fe^III^ coordination complex was utilized to block the mesopore on the sHMS to prevent leakage of the encapsulated AIPH during the circulation. Upon NIR laser irradiation, it could also act as a photothermal conversion motif to trigger the decomposition of AIPH to produce •C radical. After the nanogenerator enters in tumor cells, the intracellular overproduced GSH would disintegrate the AsHMS via disulfide linkage breaking, leading to the consuming of endogenous GSH and the explosive release of generated •C radical. Simultaneously, the released Fe^3+^ would be reduced to Fe^2+^ by TA (lysosomal acidity‐activated reducing agent), which could initiate Fention reaction to generate •OH for chemodynamic therapy. Additionally, the consumption of GSH could amplify the oxidative stress to enhance the therapeutic efficiency of •C/•OH by disrupting the self‐defense mechanism of tumor cells. Compared with conventional antitumor modalities, this work will provide a reference to develop novel oxidative stress‐based anticancer strategies.

## 2 Results and Discussion

### 2.1 Synthesis of AsHMS‐TA/Fe^III^@NK

**Figure 1:**
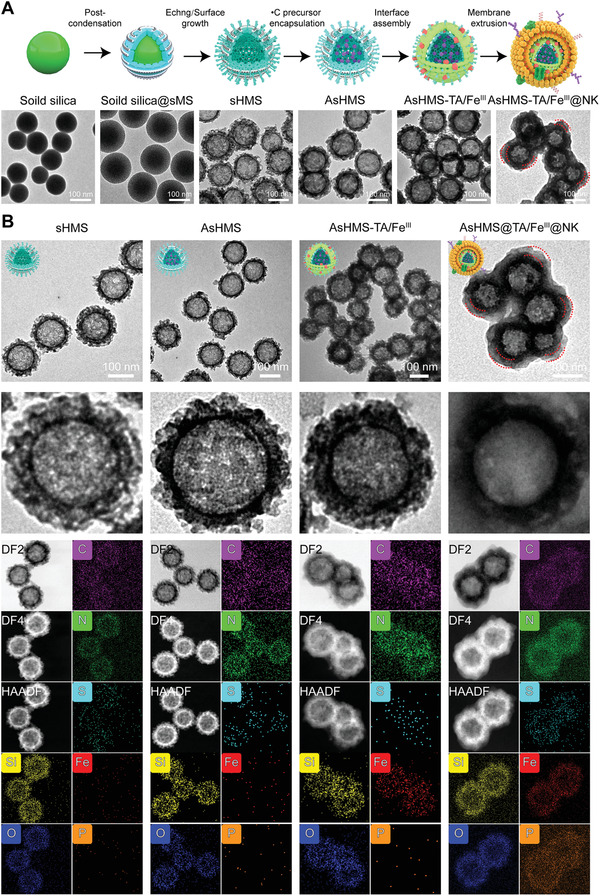
A) Synthesis routes and TEM images of AsHMS‐TA/Fe^III^@NK. B) TEM images, STEM images, EDS element mappings of sHMS, AsHMS, AsHMS‐TA/Fe^III^, and AsHMS‐TA/Fe^III^@NK.

First, the solid silica nanoparticles (**Figure** **1**A) were prepared via a modified Stöber method as previously reported.^[^ ^39^ ^]^ Subsequently, the disulfide‐doped mesoporous silica‐wrapped solid silica nanoparticles (solid silica@sMS) (Figure 1A) were prepared by post‐condensation of TEOS and BTES (bis[3‐(triethoxysilyl)propyl] disulfide, a disulfide‐doped silica precursor) (v/v, 3: 1) on the surface of solid silica nanoparticles using CTAB as a structural‐directing agent. Afterward, NaOH was utilized as an etching agent and a surface‐morphological guide agent to remove the solid silica template and produce the virus‐like disulfide‐doped hollow mesoporous nanoparticles (sHMS, the doping of disulfide bonds was determined to be ≈3.75 wt% by using a Elementar Vario EL CHNSO Elementar Analyzer). Finally, the AsHMS‐TA/Fe^III^@NK nanogenerators were prepared by encapsulation of AIPH within the hollow core, interface assembly TA/Fe^III^ on the surface, and coating NK cell membrane by extrusion. The morphology of sHMS, AsHMS, AsHMS‐TA/Fe^III^, and AsHMS‐TA/Fe^III^@NK was revealed by scanning electron microscopy (SEM) (Figure S1, Supporting Information) and transmission electron microscopy (TEM) (Figure 1), which exhibited a well‐defined hollow core‐porous shell structure, a virus‐like surface morphology, and a uniform diameter range from 100 to 150 nm (sHMS: 125.3 ± 17.8 nm; AsHMS: 129.5 ± 12.6 nm; AsHMS‐TA/Fe^III^: 131.2 ± 16.4 nm). Interestingly, the coronaviruses were nearly spherical with a diameter range from 120 to 130 nm, a “spiky” external surface, and a spike radial length of ≈20 nm, as depicted in a recent study by cryo‐electron tomography and cryo‐electron microscopy,^[^ ^40^ ^]^ which were similar to our nanoparticles with a spike radial length range from 15 to 20 nm. When the NK cell membrane was served as the “cloaking shell” for virus‐like AsHMS‐TA/Fe^III^, a nearly spherical shape was observed with a uniform diameter of 153.2 ± 16.2 nm and a membrane shell thickness of ≈10 nm. The element distribution of sHMS, AsHMS, AsHMS‐TA/Fe^III^, and AsHMS‐TA/Fe^III^@NK was further assessed by X‐ray energy‐dispersive spectrometer (EDS) element mapping (Figure 1). The sHMS and AsHMS displayed the presence of C, N, O, Si, and S elements distributed within the shell. In contrast, the Fe element was discovered in AsHMS‐TA/Fe^III^ and P element was successively discovered in AsHMS‐TA/Fe^III^@NK, indicating the successful interface assembly of TA/Fe^III^ (further supported by detailed TEM analysis (Figure S2, Supporting Information), EDS element mapping analysis (Figure S3, Supporting Information), zeta potential change (Figure S4, Supporting Information), Fourier transform infrared (FTIR) spectra (Figure S5, Supporting Information), X‐ray photoelectron spectroscopy (XPS) (Figures S6 and S7, Supporting Information), and X‐ray diffraction (XRD) spectra (Figure S8, Supporting Information)) and NK cell membrane coating.

### 2.2 Characterization of AsHMS‐TA/Fe^III^@NK

**Figure 2:**
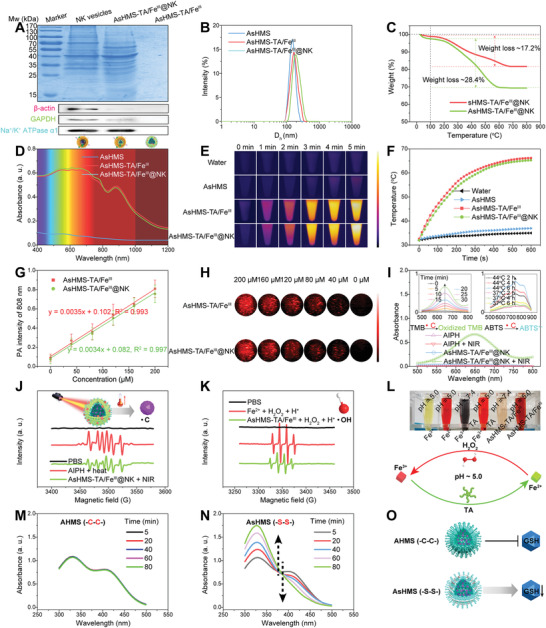
Characterization of AsHMS‐TA/Fe^III^@NK. A) SDS‐PAGE electrophoresis profiles and western blot results of AsHMS‐TA/Fe^III^@NK. NK cell membranes and AsHMS‐TA/Fe^III^ were used as controls. B) DLS profiles of AsHMS, AsHMS‐TA/Fe^III^, and AsHMS‐TA/Fe^III^@NK. C) TGA curves of sHMS‐TA/Fe^III^@NK and AsHMS‐TA/Fe^III^@NK for weight analysis of AIPH. D) UV–vis–NIR adsorption spectra of AsHMS, AsHMS‐TA/Fe^III^, and AsHMS‐TA/Fe^III^@NK. E) Thermographic images and F) temperature change curves of AsHMS, AsHMS‐TA/Fe^III^, and AsHMS‐TA/Fe^III^@NK during NIR laser irradiation (808 nm, 0.5 W cm^−2^, 5 min). G) Linear relationship between photoacoustic signals and concentrations (*n* = 3), and H) photoacoustic images of AsHMS‐TA/Fe^III^ and AsHMS‐TA/Fe^III^@NK. I) UV–vis adsorption spectra of oxidized TMB after addition of AsHMS‐TA/Fe^III^@NK into TMB with/without NIR laser irradiation. AIPH with/without NIR laser irradiation was used as the control. Inset: UV–vis spectra of oxidized TMB after the addition of AsHMS‐TA/Fe^III^@NK into TMB with NIR laser irradiation for different incubation times (left), and UV–vis spectra of ABTS^+•^ after the addition of AsHMS‐TA/Fe^III^@NK into ABTS at different incubation temperatures and times (right). J) ESR spectrum of AsHMS‐TA/Fe^III^@NK with NIR laser irradiation in the presence of DMPO. AIPH with heat treatment was used as the positive control. K) ESR spectra of AsHMS‐TA/Fe^III^ with addition of H_2_O_2_ at acidic pH in the presence of DMPO. Fe^2+^ with addition of H_2_O_2_ at acidic pH was used as the control. L) Photographs of different formulations with o‐phenanthroline in PBS at pH 5.0 or 7.4, indicative of cyclic oxidation‐reduction of Fe^3+^/Fe^2+^ under acidic condition. M, N) UV–vis spectra of DTNB as a trapping agent of sulphydryl (—SH) in GSH after addition of M) AHMS‐TA/Fe^III^@NK and N) AsHMS‐TA/Fe^III^@NK, indicative of the GSH depleting ability. O) Schematic illustration of GSH consumption by GSH‐responsive AsHMS‐TA/Fe^III^ (—S—S—). GSH‐irresponsive AHMS‐TA/Fe^III^ (—C—C—) was used as the control.

The membrane components on NK cells played a vital role in their functions including evading immune clearance.^[^ ^41^, ^42^ ^]^ The protein profile of AsHMS‐TA/Fe^III^@NK was analyzed by the SDS‐PAGE electrophoresis assay (**Figure** **2**A). The results indicated that the complexity of NK cell membrane was successfully preserved, enabling the AsHMS‐TA/Fe^III^@NK to retain the original activity of NK cells. The Western blot results showed that some membrane protein components including Na^+^/K^+^ ATPase*α*1 were identically transferred from NK cells to AsHMS‐TA/Fe^III^@NK (Figure 2A). Dynamic light scattering results indicated that the hydrodynamic diameter was slightly increased in AsHMS‐TA/Fe^III^@NK after TA/Fe^III^ interface assembly and NK cell membrane coating (Figure 2B). In virtue of the mesoporous shell structure and large hollow cavity of sHMS, AIPH can be easily encapsulated within sHMS. XRD spectra (Figure S8, Supporting Information), N_2_ adsorption‐desorption isotherms (Figure S9, Supporting Information), and pore diameter distributions (Figure S10, Supporting Information) indicated the successful loading of AIPH molecules within the pore channels of sHMS. Thermogravimetric analysis (TGA) was performed to quantify the weight of AIPH molecules in the nanogenerators. By determining the variation of weight loss between sHMS‐TA/Fe^III^@NK and AsHMS‐TA/Fe^III^@NK at 700 °C (Figure 2C), the loading efficiency of AIPH within AsHMS‐TA/Fe^III^@NK was calculated to be ≈11.2%.

### 2.3 Photothermal and Photoacoustic Performance

UV–vis–NIR absorption spectra showed that AsHMS‐TA/Fe^III^ and AsHMS‐TA/Fe^III^@NK both exhibited strong absorption with a unique absorption peak at ≈880 nm in the NIR window (Figure 2D). This phenomenon encouraged us to further investigate the photothermal conversion capacity of AsHMS‐TA/Fe^III^@NK nanogenerators (Figure 2E,F). Temperature change curves and thermographic images demonstrated the concentration‐dependent temperature increase behavior and the high photothermal conversion ability of both AsHMS‐TA/Fe^III^ and AsHMS‐TA/Fe^III^@NK. Particularly, the temperature of AsHMS‐TA/Fe^III^ and AsHMS‐TA/Fe^III^@NK dramatically increased from ≈32.0 to ≈65.3 and 66.2 °C at 100 µg mL^−1^ of sHMS concentration during 808 nm laser irradiation (0.5 W cm^−2^, 5 min), whereas the temperature of AsHMS just increased to ≈36.8 °C under the same condition. After verifying the excellent photothermal potential of AsHMS‐TA/Fe^III^@NK, the photoacoustic capabilities were further assessed. As presented in Figure 2G,H, both AsHMS‐TA/Fe^III^ and AsHMS‐TA/Fe^III^@NK possessed comparable photoacoustic signals, which was consistent with the UV–vis absorption results. At the wavelength of 808 nm, the photoacoustic intensity values of both AsHMS‐TA/Fe^III^ and AsHMS‐TA/Fe^III^@NK were linearly related to their concentrations (Figure 2G).

### 2.4 •C Radical Generation Ability

Inspired by the excellent photothermal conversion capacity of AsHMS‐TA/Fe^III^@NK, we further explored the •C radical generation ability of AsHMS‐TA/Fe^III^@NK exposed to laser‐induced hyperthermia by determining the characteristic absorbance of oxidized 3,3′,5,5′‐tetramethylbenzidine (oxidized TMB, reaction production between TMB and generated •C radical) and 2,2′‐azinobis(3‐ethylbenzothiazoline‐6‐sulfonic acid) (ABTS^+•^, reaction production between ABTS and generated •C radical), respectively (Figure 2I). After NIR laser irradiation, the characteristic absorbance of oxidized TMB at ≈650 nm in AsHMS‐TA/Fe^III^@NK gradually increased in a time‐dependent manner, which was remarkably different with the AIPH, AIPH plus NIR laser irradiation, and AsHMS‐TA/Fe^III^@NK groups. With the increased temperature and extended time, the generation of oxidative TMB was also increased (inset of Figure 2I, left). The absorbance of ABTS^+•^ in the range of 500–900 nm was also significantly higher at 44 °C comparing to that at 37 °C (inset of Figure 2I, right), indicating the faster decomposition of APIH and release of •C radical at the elevated temperature. As displayed in Figure 2J, the •C radical was observed from the sextet signals in the electron spin‐resonance spectroscopy (ESR) spectrum of AsHMS‐TA/Fe^III^@NK plus NIR laser irradiation or AIPH plus heat treatment. These findings indicated their potential energy conversion ability for photothermal‐primed thermodynamic therapy.

### 2.5 •OH Radical Generation Ability

It was reported that natural polyphenol TA could be utilized as an acid‐activated reducing agent.^[^ ^43^ ^]^ Thus TA might converse Fe^3+^ into Fe^2+^ in the highly acidic organelles of tumor cells (e.g., endo/lysosomes), which could initiate Fenton reaction in the presence of intracellular overproduced H_2_O_2_, thereby generating •OH radical. To verify this, the •OH radical generation ability of AsHMS‐TA/Fe^III^ with addition of H_2_O_2_ at acidic pH was investigated. As displayed in Figure 2K, the •OH radical was observed from the characteristic 1: 2: 2: 1 quartet signals in the ESR spectrum of AsHMS‐TA/Fe^III^ plus H^+^/H_2_O_2_ group or Fe^2+^ plus H^+^/H_2_O_2_ group. The result was further proved by an o‐phenanthroline assay (Figure 2L), XPS high‐resolution spectra of Fe2p region (Figures S11–S13, Supporting Information), and methylene blue (MB) oxidation assay (Figure S14, Supporting Information). O‐phenanthroline could form orange complexes with Fe^2+^ but not Fe^3+^. By observing the color changes, pH 5.0 was more powerful for conversing Fe^3+^ into Fe^2+^ for AsHMS‐TA/Fe^III^ compared with pH 7.4 (Figure 2L). By comparison with the AsHMS‐TA/Fe^III^, the binding energy of AsHMS‐TA/Fe^III^ after acidic treatment (pH 5.0) in XPS high‐resolution spectra of Fe2p region shifted to the lower binding energy side (Fe2p3/2 (Fe^III^) 712.0 eV→Fe2p3/2 (Fe^II^) 710.2 eV; Fe2p1/2 (Fe^III^) 726.0 eV→Fe2p1/2 (Fe^II^) 723.4 eV) (Figure S13, Supporting Information). We also found that MB was oxidized in AsHMS‐TA/Fe^III^ plus H^+^/H_2_O_2_ group rather than in AsHMS plus H^+^/H_2_O_2_ group (Figure S14, Supporting Information). These results proved the conversion of Fe^3+^ into Fe^2+^ by TA under acidic condition. These findings indicated that the nanogenerators possessed excellent acidity responsiveness and high •OH radical generation ability for “Fe^3+^/Fe^2+^” circular amplification via cyclic oxidation‐reduction (Figure 2L) and continuous chemodynamic therapy.

### 2.6 GSH Depletion Ability

The overproduced GSH in tumor cells as an antioxidant would directly participate in the elimination of free radicals to keep redox homeostasis, thus decreasing the therapeutic efficiency of free radical‐based therapy.^[^ ^23^, ^44^, ^45^ ^]^ As AsHMS‐TA/Fe^III^ could trigger the disintegration of nanogenerators via disulfide‐thiol exchange by GSH stimulus, we wondered whether the synthesized AsHMS‐TA/Fe^III^ nanogenerators possessed the GSH depleting ability. After treating AsHMS‐TA/Fe^III^ with an excess of GSH in different treatment times, the residual GSH could be determined via a 5,5′‐dithiobis (2‐nitrobenzoic acid) (DTNB, a —SH indicator) assay. The GSH‐irresponsive AHMS‐TA/Fe^III^ was utilized as the control. With the extension of treatment time, the characteristic absorbance of DTNB gradually decreased at ≈410 nm in AsHMS‐TA/Fe^III^ but not in AHMS‐TA/Fe^III^, implying that AsHMS‐TA/Fe^III^ nanogenerators depleted the GSH via thiol‐disulfide exchange reaction (Figure 2M–O). In addition, the intracellular GSH/GSSG ratio after incubation of cells with nanogenerators was detected by using the GSH/GSSG‐Glo Assay Kit. As seen in Figure S15, Supporting Information, the GSH/GSSG ratio in sHMS‐treated cancer cells was greatly declined from ≈15 to ≈6 compared with PBS‐treated cells, which confirmed that GSH could be depleted by sHMS via disulfide–thiol exchange.

### 2.7 NIR‐Triggered Shape Reversal of AsHMS‐TA/Fe^III^@NK and GSH‐Triggered Disassembly of AsHMS‐TA/Fe^III^

**Figure 3:**
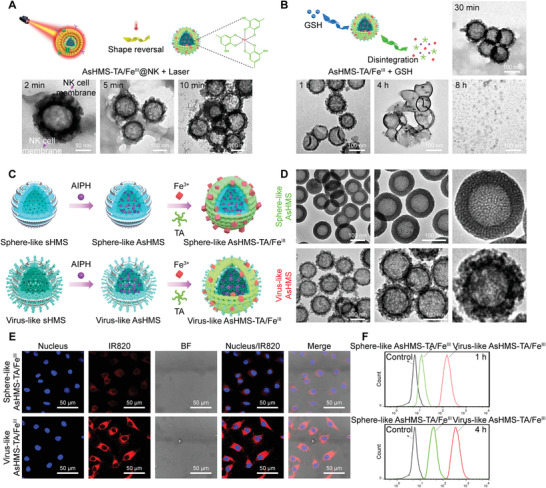
NIR‐triggered shape reversal of AsHMS‐TA/Fe^III^@NK and GSH‐triggered disassembly of AsHMS‐TA/Fe^III^. A) Schematic illustration of AsHMS‐TA/Fe^III^@NK and Time‐lapsed TEM images of AsHMS‐TA/Fe^III^@NK with NIR laser irradiation (808 nm, 0.5 W cm^−2^, 5 min). B) Schematic illustration and Time‐lapsed TEM images of AsHMS‐TA/Fe^III^@NK with initial NIR laser/subsequent GSH stimulus. Comparison of virus‐like AsHMS‐TA/Fe^III^ nanogenerators versus sphere‐like AsHMS‐TA/Fe^III^ nanogenerators. C) Schematic illustration of synthesis and D) TEM images of sphere‐like and virus‐like nanogenerators. E) CLSM images and F) flow cytometric profiles of HepG2 cells incubated with sphere‐like and virus‐like nanogenerators for 4 h.

Inspired by the excellent photothermal performance, the morphology change was also observed using TEM to verify the hyperthermia‐induced cell membrane rupture of AsHMS‐TA/Fe^III^@NK nanogenerators upon NIR laser irradiation. When the AsHMS‐TA/Fe^III^@NK was exposed to NIR laser irradiation (0.5 W cm^−2^, 5 min) and observed after 10 min, the virus‐like surface was found to be re‐exposed in the AsHMS‐TA/Fe^III^@NK nanogenerators (**Figure** **3**A; Figures S2 and S3, Supporting Information). This result proved the AsHMS‐TA/Fe^III^@NK could response to the NIR laser stimulus for shape switch. To further investigate the GSH responsiveness of AsHMS‐TA/Fe^III^, the AsHMS‐TA/Fe^III^ nanogenerators were incubated with 5 mm of GSH for 8 h. TEM images indicated that the AsHMS‐TA/Fe^III^ kept its morphological integrity in PBS buffer without GSH within 4 h. Comparatively, the AsHMS‐TA/Fe^III^ underwent rapid morphological transformation after dispersion in PBS buffer with adding 5 mm GSH during the same period (Figure 3B). After 8 h, the AsHMS‐TA/Fe^III^ almost completely collapsed in PBS buffer with GSH adding. These findings demonstrated the high GSH sensitivity of AsHMS‐TA/Fe^III^, conferring it with high specificity for the disintegration and sequential on‐demand release of generated •C radical in high GSH environment.

### 2.8 Comparison of Virus‐Like AsHMS‐TA/Fe^III^ Nanogenerators versus Sphere‐Like AsHMS‐TA/Fe^III^@NK Nanogenerators

To achieve effective thermodynamic–chemodynamic therapy, before the rapid release of •C/•OH radical, the nanogenerators should efficiently enter cancer cells. Thus we used a fluorescent dye IR820‐NHS to label the sHMS to investigate the cell entry. In consideration of the similar hydrodynamic particle size and surface charge of virus‐like and sphere‐like AsHMS, the sphere‐like AsHMS could be selected as a control (Figure 3C,D, and Figure S16, Supporting Information). By confocal laser scanning microscopy (CLSM) observation, the significantly stronger red fluorescence was observed in virus‐like AsHMS‐TA/Fe^III^‐treated HepG2 cells for 4 h compared to sphere‐like AsHMS‐TA/Fe^III^‐treated cells (Figure 3E). By flow cytometry, the red fluorescence in virus‐like AsHMS‐TA/Fe^III^‐treated HepG2 cells was found to be approximately ninefold and approximately fivefold higher than the sphere‐like AsHMS‐TA/Fe^III^‐treated cells for 1 and 4 h, respectively (Figure 3F and Figure S17, Supporting Information). Theoretically, the virus‐like nanoparticles exhibited more contact sites per unit area with cell membranes compared to sphere‐like nanoparticles, which was beneficial to increase the adhesive interaction.^[^ ^24^, ^25^ ^]^ Both qualitative and quantitative results indicated that the introduction of a virus‐like rough surface could significantly improve the cell entry efficiency.

### 2.9 In Vitro Cell Entry and Macrophage Phagocytosis

**Figure 4:**
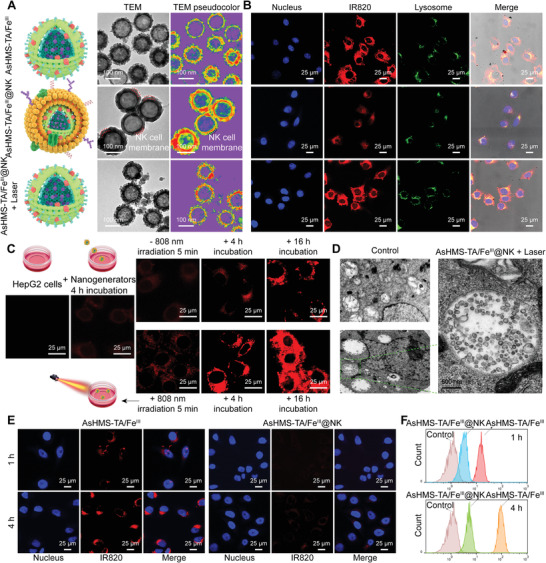
In vitro cell entry and macrophage phagocytosis. A) SEM images, TEM images, TEM pseudocolor images of AsHMS‐TA/Fe^III^ and AsHMS‐TA/Fe^III^@NK nanogenerators. B) CLSM images of HepG2 cells incubated with AsHMS‐TA/Fe^III^ and AsHMS‐TA/Fe^III^@NK with/without NIR laser irradiation (808 nm, 0.5 W cm^−2^, 5 min; the highest temperature for cell entry of nanogenerators with NIR laser irradiation was determined to be ≈47 °C). C) Detailed CLSM images of HepG2 cells incubated with AsHMS‐TA/Fe^III^@NK nanogenerators with/without NIR laser irradiation (808 nm, 0.5 W cm^−2^, 5 min) for different incubation times. D) Cellular TEM images of HepG2 cells incubated with AsHMS‐TA/Fe^III^ with NIR laser irradiation (808 nm, 0.5 W cm^−2^, 5 min). E) CLSM images and F) flow cytometric profiles of macrophage RAW 264.7 cells incubated with AsHMS‐TA/Fe^III^ and AsHMS‐TA/Fe^III^@NK nanogenerators for 1 and 4 h.

HepG2 cells were further treated with AsHMS‐TA/Fe^III^@NK with and without NIR laser irradiation, and AsHMS‐TA/Fe^III^ was used as the control. The fluorescence signals of AsHMS‐TA/Fe^III^@NK‐treated HepG2 cells were starkly decreased compared to those of AsHMS‐TA/Fe^III^‐treated cells, which was likely due to the NK cell membrane cloaking for virus‐like rough surface shielding (**Figure** **4**A,B). Moreover, when the AsHMS‐TA/Fe^III^@NK was exposed to NIR laser irradiation (808 nm, 0.5 W cm^−2^, 5 min), the intracellular fluorescence signals could be dramatically increased due to the re‐exposure of virus‐like rough surface via NIR laser‐triggered NK cell membrane rupture, and these signals were very close to those of the original AsHMS‐TA/Fe^III^‐treated cells (Figure 4A,B). We also performed the cellular uptake of “sphere‐like AsHMS‐TA/Fe^III^”@NK, “virus‐like AsHMS‐TA/Fe^III^”@NK, sphere‐like AsHMS‐TA/Fe^III^, and virus‐like AsHMS‐TA/Fe^III^ with and without NIR laser irradiation (808 nm, 0.5 W cm^−2^, 5 min) (Figures S18 and S19, Supporting Information). Compared with the AsHMS‐TA/Fe^III^ (sphere‐like or virus‐like) without NIR laser irradiation, the AsHMS‐TA/Fe^III^ with NIR laser irradiation slightly enhanced the cellular uptake. More significantly, compared with the “sphere‐like AsHMS‐TA/Fe^III^”@NK with NIR laser irradiation, the “virus‐like AsHMS‐TA/Fe^III^”@NK with NIR laser irradiation remarkably elevated the cellular uptake. Therefore, we believed that after NIR laser irradiation, both factors including the restoration of virus‐mimicking surface feature and the temperature increase‐induced cell membrane destabilization could promote the cell entry: the former factor played a major role while the latter factor also should be not ignored. CLSM observation and flow cytometry analysis for extended incubation times further proved the NIR laser‐triggered elevation of cell entry (Figure 4C and Figure S20, Supporting Information). In addition, the cellular ultrastructure observed by TEM showed that the virus‐like AsHMS‐TA/Fe^III^ was entered inside the intracellular lysosomal space (Figure 4D), which was correlated well with the co‐localization of AsHMS‐TA/Fe^III^ fluorescence and LysoTracker Green fluorescence observed by CLSM (Figure 4B), implying the cell entry of virus‐mimicking nanogenerators via endocytotic pathway. These results proved that the NIR laser‐responsive shape reversal could endow AsHMS‐TA/Fe^III^@NK with high tumor selectivity to realize the elevated cell entry in the tumor microenvironment.

To investigate the immune‐evading ability of AsHMS‐TA/Fe^III^@NK, the macrophage RAW 264.7 cells were treated with AsHMS‐TA/Fe^III^@NK for 1 and 4 h, and the AsHMS‐TA/Fe^III^ was used as the control. The red fluorescence inside AsHMS‐TA/Fe^III^@NK‐treated cells was significantly weaker compared to that of AsHMS‐TA/Fe^III^‐treated cells (Figure 4E), indicating the prominently decreased macrophage uptake of AsHMS‐TA/Fe^III^@NK after the cloaking of NK cell membrane. The flow cytometry analysis supported the CLSM observation (Figure 4F and Figure S21, Supporting Information). The finding was likely due to the fact that the shielding of surface roughness by NK cell membrane could effectively reduce the macrophage recognition and uptake. To further investigate the immune‐evading ability of AsHMS‐TA/Fe^III^@NK, the secretions of TNF‐*α* and IL‐10 were determined by an ELISA kit. The secretion of TNF‐*α* and IL‐10 of macrophage RAW 264.7 cells treated with AsHMS‐TA/Fe^III^@NK was significantly decreased compared to that of cells treated with AsHMS‐TA/Fe^III^ (Figure S22, Supporting Information), indicating that the immune responses of AsHMS‐TA/Fe^III^@NK were far lower than AsHMS‐TA/Fe^III^. These findings proved that the “virus‐to‐sphere” shape transformation with NK cell membrane shielding could evade the macrophage recognition and uptake to improve the immune‐evading ability.

### 2.10 In Vitro Anticancer Efficacy

**Figure 5:**
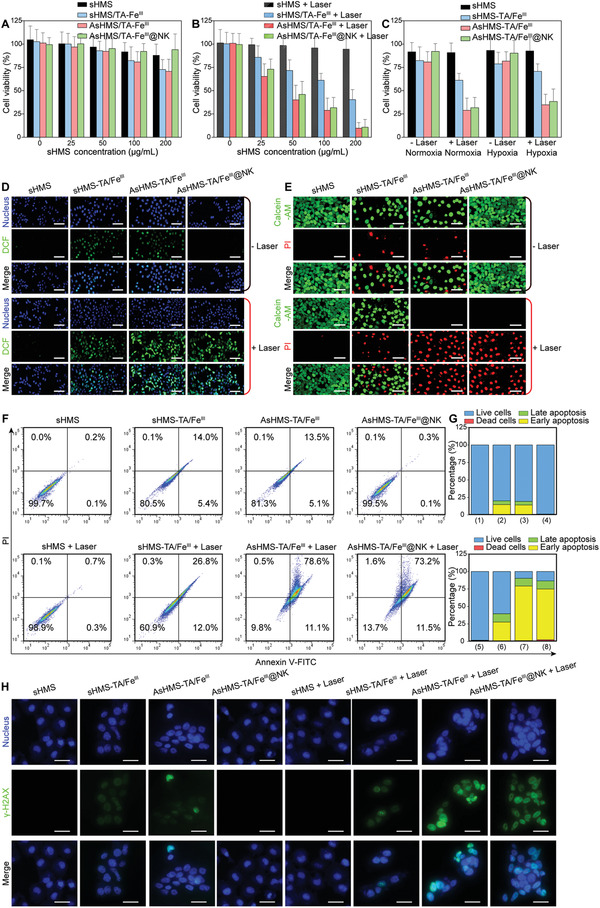
In vitro anticancer efficiency. A, B) Viability of HepG2 cells treated with sHMS, sHMS‐TA/Fe^III^, AsHMS‐TA/Fe^III^, AsHMS‐TA/Fe^III^@NK for 24 h with/without NIR laser irradiation (0.5 W cm^−2^, 5 min) at different sHMS concentrations (*n* = 4). C) Viability of HepG2 cells treated with sHMS, sHMS‐TA/Fe^III^, AsHMS‐TA/Fe^III^, AsHMS‐TA/Fe^III^@NK for 24 h with/without NIR laser irradiation (0.5 W cm^−2^, 5 min) at sHMS concentration of 100 µg mL^−1^ (*n* = 4). D) Intracellular free radical generation via DCFH‐DA assay, E) live/dead cell staining via calcein‐AM/PI assay, and F,G) apoptosis assay via Annexin V‐FITC/PI staining, H) Intracellular DNA double‐strand breakage via *γ*‐H_2_AX assay in HepG2 cells treated with sHMS, sHMS‐TA/Fe^III^, AsHMS‐TA/Fe^III^, and AsHMS‐TA/Fe^III^@NK for 6 h with/without NIR laser irradiation (0.5 W cm^−2^, 5 min).

To assess the in vitro therapeutic efficacy, CCK‐8 assays were executed to determine the cell viability. First, different cell lines (normal HUVEC cells; carcinoma 4T1, MCF‐7, and HepG2 cells) were incubated with AsHMS‐TA/Fe^III^@NK in the dark for 24 or 48 h, and no significant decrease in cell viability was observed even when the sHMS concentration was increased to 500 µg mL^−1^ (Figures S23 and S24, Supporting Information), indicating the negligible cytotoxicity of AsHMS‐TA/Fe^III^@NK. Without NIR laser irradiation, the sHMS‐TA/Fe^III^ and AsHMS‐TA/Fe^III^‐treated HepG2 cells exhibited a partial cytotoxicity, which could be explained by the exerting chemodynamic effect of TA/Fe^III^ coordination (**Figure** **5**A). Under NIR laser irradiation (0.5 W cm^−2^, 5 min), the sHMS possessed almost no effect on cytotoxicity (≈90.9% of cell viability) with the concentration of 100 µg mL^−1^, but sHMS‐TA/Fe^III^ caused the moderate cytotoxicity (≈61.1% of cell viability), and the AsHMS‐TA/Fe^III^ induced the most severe cytotoxicity (≈28.9% of cell viability) at equivalent sHMS concentration due to the photothermal‐primed thermodynamic–chemodynamic effect (Figure 5B). Notably, after exposure to NIR laser, the AsHMS‐TA/Fe^III^@NK‐treated HepG2 cells displayed a comparable cytotoxicity with the AsHMS‐TA/Fe^II^‐treated cells (Figure 5C), which was due to the NIR laser‐triggered shape transformation of AsHMS‐TA/Fe^III^@NK to re‐expose the virus‐like surface for elevated cell entry. In addition, under NIR laser irradiation, the AsHMS‐TA/Fe^III^@NK‐treated HepG2 cells exhibited a comparable cytotoxicity under both normoxia and hypoxia conditions (Figure 5C), indicating that the generation of •C/•OH radical was independent of intracellular oxygen level.

To discover whether AsHMS‐TA/Fe^III^ could act as intracellular nanogenerators to generate •C/•OH radical, 2′,7′‐dichlorodihydrofluorescein diacetate (DCFH‐DA) was utilized to directly visualize the intracellular radical levels. As shown in Figure 5D, the brightest green fluorescence emitted from DCF was observed in AsHMS‐TA/Fe^III^ and AsHMS‐TA/Fe^III^@NK plus NIR laser irradiation compared with the other groups, indicating the more significant intracellular free radical generation and meaning more effective thermodynamic–chemodynamic effect of AsHMS‐TA/Fe^III^@NK with NIR laser irradiation. The live/dead cell staining result via calcein‐AM/PI assay (Figure 5E) and the apoptosis result via Annexin V‐FITC/propidium iodide (PI) assay (Figure 5F,G and Figure S25, Supporting Information) further demonstrated that photothermal‐primed thermodynamic–chemodynamic therapy induced highly efficient cancer cell‐killing via apoptosis/death pathway. In addition, the highly reactive •C radical tend to electrophilic addition reactions with intracellular bioactive molecules (e.g., DNA), to inhibit DNA replication/transcription by one‐electron oxidation of DNA.^[^ ^46^, ^47^ ^]^ To prove the DNA damage in tumors, the immunofluorescence analysis of *γ*‐H_2_AX as a biomarker of DNA double‐strand breakage was carried out. The AsHMS‐TA/Fe^III^ + Laser and AsHMS‐TA/Fe^III^@NK + Laser groups exhibited strong green fluorescent signals in HepG2 cells, whereas significantly weaker green fluorescent signals were observed in the other groups (Figure 5H), indicating the AsHMS‐TA/Fe^III^@NK nanogenerators in tumor cells under NIR irradiation effectively induced intracellular DNA double‐strand breakage.

### 2.11 In Vivo Biodistribution and Pharmacokinetics

**Figure 6:**
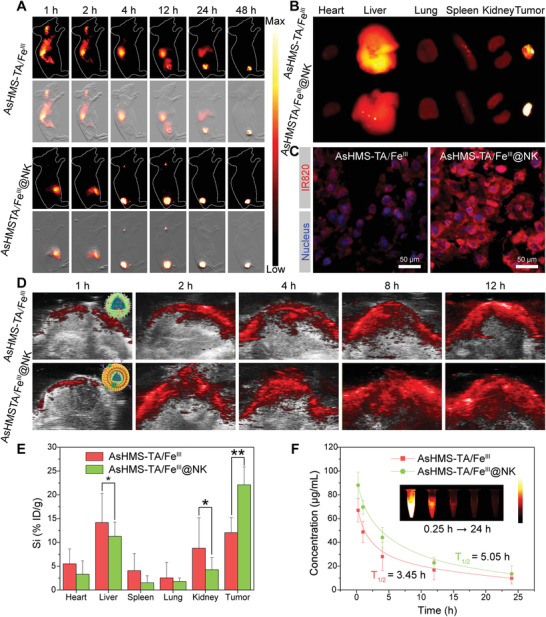
In vivo biodistribution and pharmacokinetics. A) Time‐lapsed NIR‐II fluorescence imaging of HepG2 tumor‐bearing nude mice after intravenous injection of IR820‐labeled AsHMS‐TA/Fe^III^ and AsHMS‐TA/Fe^III^@NK. B) NIR‐II fluorescence imaging of excised normal and tumor tissues at 48 h post‐injection. C) Intracellular fluorescence of AsHMS‐TA/Fe^III^ and AsHMS‐TA/Fe^III^@NK in excised tumor tissues at 48 h post‐injection. D) Time‐lapsed photoacoustic imaging in tumor tissues of HepG2 tumor‐bearing nude mice after intravenous injection of AsHMS‐TA/Fe^III^ and AsHMS‐TA/Fe^III^@NK. E) Si element biodistribution and F) pharmacokinetics of HepG2 tumor‐bearing nude mice after intravenous injection of AsHMS‐TA/Fe^III^ and AsHMS‐TA/Fe^III^@NK (*n* = 4). *p < 0.05; **p < 0.01.

To investigate the biodistribution of AsHMS‐TA/Fe^III^@NK, the sHMS was labeled with IR820‐NHS. After intravenous injection in HepG2 tumor‐bearing nude mice, the significant NIR‐II fluorescence signals could be observed in the tumor areas of the AsHMS‐TA/Fe^III^@NK group within 4 h (**Figure** **6**A and Figure S26A, Supporting Information). In contrast, few NIR‐II fluorescence signals were found in the AsHMS‐TA/Fe^III^ group at the same time. Additionally, the semi‐quantitative analysis showed that the NIR‐II fluorescence signal of excised tumor tissues in the AsHMS‐TA/Fe^III^@NK group was ≈1.75‐fold than that of AsHMS‐TA/Fe^III^ group (Figure 6B and Figure S26B, Supporting Information), which was in accordance with the frozen section results by CLSM observation (Figure 6C).

Then, NIR photoacoustic imaging was further performed to verify the tumor accumulation of AsHMS‐TA/Fe^III^@NK. After intravenous injection of AsHMS‐TA/Fe^III^ and AsHMS‐TA/Fe^III^@NK, the photoacoustic signals of HepG2 tumor bearing mice were recorded at different time points at the tumor site. The photoacoustic signals at the tumor site increased gradually and reached the highest level at 12 h post‐injection (Figure 6D and Figure S27, Supporting Information). Moreover, the AsHMS‐TA/Fe^III^@NK group exhibited significantly enhanced photoacoustic signals in the tumor tissues compared to AsHMS‐TA/Fe^III^ group. Furthermore, after 24 h of injection, by using ICP‐OES to determine Si element, the biodistribution of AsHMS‐TA/Fe^III^ and AsHMS‐TA/Fe^III^@NK showed a similar trend (Figure 6E). In addition, the circulation lifetime of AsHMS‐TA/Fe^III^@NK (half‐life time: ≈5.05 h) was significantly prolonged over that of AsHMS‐TA/Fe^III^ (half‐life time: ≈3.45 h) (Figure 6F), which also explained the highly effective tumor accumulation of AsHMS‐TA/Fe^III^@NK over AsHMS‐TA/Fe^III^.

### 2.12 In Vivo Therapeutic Effect

**Figure 7:**
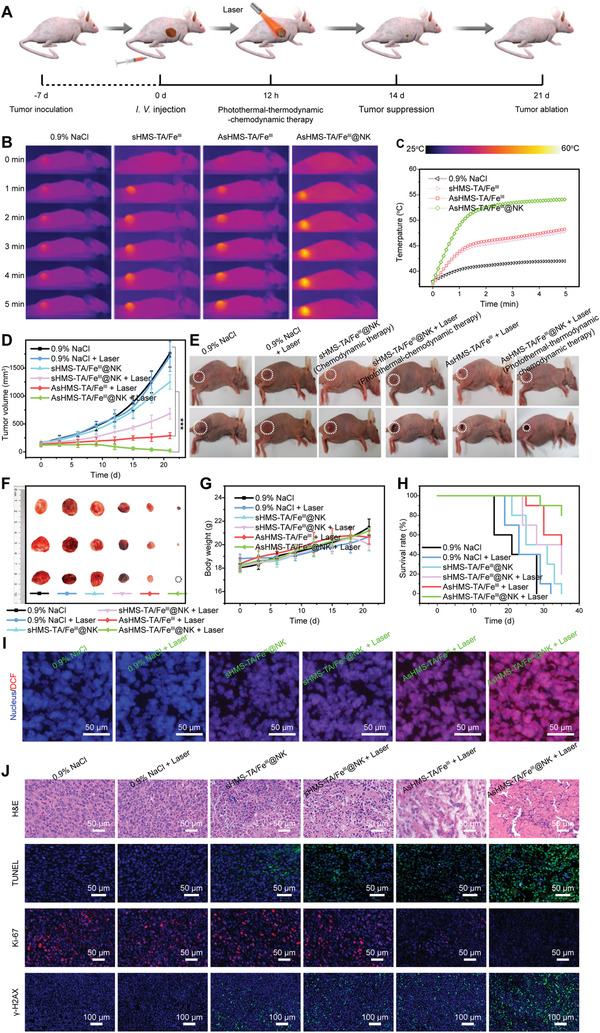
In vivo therapeutic effect. A) Illustration of establishment and treatment schedule of HepG2 tumor‐bearing nude mice model. B) Thermographic images of HepG2 tumor‐bearing nude mice and C) relevant temperature change curves of tumors after intravenous injection of 0.9% NaCl, sHMS‐TA/Fe^III^@NK, AsHMS‐TA/Fe^III^, AsHMS‐TA/Fe^III^@NK during NIR laser irradiation (808 nm, 0.5 W cm^−2^, 5 min). D) Tumor growth curves (*n* = 10), E) tumor growth photographs, F) excised tumor photographs, G) body weights (*n* = 10), and H) survival rate (*n* = 10) of HepG2 tumor‐bearing nude mice after intravenous injection of 0.9% NaCl, sHMS‐TA/Fe^III^@NK, AsHMS‐TA/Fe^III^, AsHMS‐TA/Fe^III^@NK with/without NIR laser irradiation. I) DCFH‐DA‐stained tumor slices and J) H&E/TUNEL/Ki‐67/*γ*‐H_2_AX antibody‐stained tumor slices of HepG2 tumor‐bearing nude mice after intravenous injection of 0.9% NaCl, sHMS‐TA/Fe^III^@NK, AsHMS‐TA/Fe^III^, AsHMS‐TA/Fe^III^@NK with/without NIR laser irradiation. ***p < 0.001. The sHMS‐TA/Fe^III^@NK, sHMS‐TA/Fe^III^@NK + Laser, AsHMS‐TA/Fe^III^@NK+ Laser group represented chemodynamic therapy, photothermal–chemodynamic therapy, photothermal–thermodynamic–chemodynamic therapy, respectively.

The therapeutic efficacy of nanogenerators in vivo was further assessed. After the maximizing tumor accumulation of AsHMS‐TA/Fe^III^@NK at 12 h post‐injection, NIR laser was irradiated at the tumor sites (**Figure** **7**A). First, the thermographic images of mice injected with AsHMS‐TA/Fe^III^ and AsHMS‐TA/Fe^III^@NK were acquired (Figure 7B,C). The temperature in the tumor areas of mice treated with AsHMS‐TA/Fe^III^@NK rapidly increased to ≈54.1 °C. In contrast, the temperature in the tumor areas of mice treated with sHMS‐TA/Fe^III^ and AsHMS‐TA/Fe^III^ increased to ≈47.8 and ≈48.3 °C under the same condition, respectively. The result suggested that better photothermal effects of AsHMS‐TA/Fe^III^@NK upon NIR laser irradiation were achieved.

The tumor size of HepG2 tumor‐bearing nude mice was recorded every 3 days to estimate the therapeutic effect. As shown in Figure 7D,E, the rapid tumor growth was observed in mice treated with 0.9% NaCl, 0.9% NaCl plus NIR laser irradiation, and sHMS‐TA/Fe^III^@NK (chemodynamic therapy) during 21‐day treatment, and the moderate tumor growth was appeared in mice treated with sHMS‐TA/Fe^III^@NK plus NIR laser irradiation (photothermal–chemodynamic therapy). In contrast, the AsHMS‐TA/Fe^III^ treatment plus NIR laser irradiation and AsHMS‐TA/Fe^III^@NK treatment plus NIR laser irradiation (photothermal–thermodynamic–chemodynamic therapy) produced significant tumor growth inhibition in mice with tumor inhibition rates of 86.3% and 98.9%, respectively. Particularly, the major of tumors started to shrank dramatically on the 6th day, and even some tumors were completely eliminated on the 21st day in AsHMS‐TA/Fe^III^@NK + Laser group, indicating AsHMS‐TA/Fe^III^@NK possessed a superior therapeutic effect via the synergy of photothermal, thermodynamic, and chemodynamic therapy. Additionally, the relevant excised tumor photographs at the end of treatment showed similar antitumor trends (Figure 7F and Figure S28, Supporting Information). Furthermore, DCFH‐DA, hematoxylin and eosin (H&E), TUNEL, Ki‐67, and*γ*‐H_2_AX antibody staining of tumor tissues was performed to estimate the histopathological changes. The highest intratumoral ROS levels, the largest necrosis area with the most serious nuclear condensation, the most extensive apoptotic nuclear fragmentation, the lowest Ki‐67 antigen expression, and the most DNA double‐strand breakage^[^ ^47^ ^]^ were reflected in the tumors of mice treated with AsHMS‐TA/Fe^III^@NK plus NIR laser irradiation (Figure 7I,J). The findings proved the superior therapeutic effects of AsHMS‐TA/Fe^III^@NK under NIR laser irradiation.

**Figure 8:**
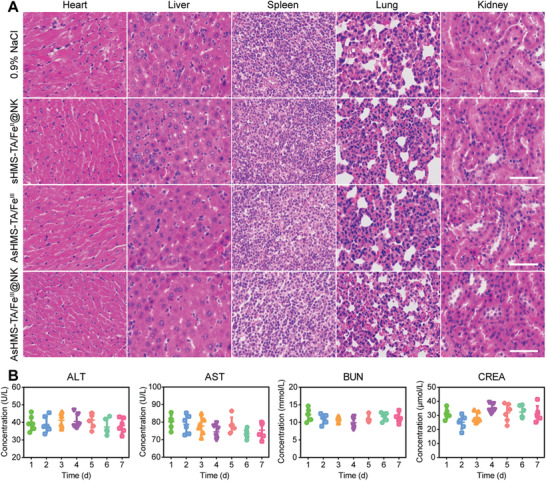
In vivo safety. A) H&E staining images of major organs from HepG2 tumor‐bearing nude mice after different treatments including 0.9% NaCl, sHMS‐TA/Fe^III^@NK, AsHMS‐TA/Fe^III^, and AsHMS‐TA/Fe^III^@NK. B) Liver function (ALT and AST levels) and kidney function (BUN and CREA levels) of HepG2 tumor‐bearing nude mice after treatment with AsHMS‐TA/Fe^III^@NK.

The body weight was also monitored every 3 days, and no distinct body weight loss was found in all groups during treatment (Figure 7G). Moreover, the survival rates of AsHMS‐TA/Fe^III^@NK+ Laser group were far above the other groups (Figure 7H). Besides, no significant histopathological abnormalities were found in the major organs of mice at the end of treatment (**Figure** **8**A). In addition, no distinct variations were detected in the blood biochemical indexes of mice with AsHMS‐TA/Fe^III^@NK treatment during 7 days (Figure 8B). These results indicated the negligible systemic toxicity of AsHMS‐TA/Fe^III^@NK during the treatment. Overall, the excellent therapeutic effects of AsHMS‐TA/Fe^III^@NK plus NIR laser irradiation could be attributed to the synergy of photothermal–thermodynamic–chemodynamic therapy via NIR‐triggered shape reversal and NIR/GSH‐responsive •C/•OH radical release, which showed the clinical translation potential.

## 3 Conclusions

In conclusion, a cytomembrane‐cloaked virus‐mimicking and NIR/GSH sequentially activated free radical nanogenerator (AsHMS‐TA/Fe^III^@NK) was constructed by wrapping the NK cell membrane on virus‐like disulfide‐doped hollow mesoporous silicon core (sHMS), which encapsulated •C initiator AIPH and then was capped by TA/Fe^III^ coordination. Upon NIR and GSH sequential triggering, the nanogenerator would realize shape reversal to rapidly invade tumor cells and in situ controllably produce •C/•OH via thermal decomposition of AIPH and TA/Fe^III^‐mediated Fenton reaction accompanied by the oxidative stress amplification to achieve photothermal‐augmented thermodynamic–chemodynamic therapy. The synergistic therapeutic effect of hyperthermia and •C/•OH radical were proved via significantly enhanced cell apoptosis in vitro and inhibiting tumor growth in vivo. This cytomembrane‐cloaked virus‐mimicking and NIR/GSH sequentially activated free radical nanogenerator could expand the strategies of oxidative stress‐based anticancer therapy.

## 4 Experimental Section

### Synthesis of Silica Templates

The solid silica nanoparticles were first prepared via a modified Stöber method.^[^ ^39^ ^]^ 1 mL of TEOS and 0.4 mL of NH_3_.H_2_O were injected into 22 mL of ethanol/water mixture (20: 2, v/v). After stirring for 10 h, the products were collected via centrifugation (8000 rpm, 0.5 h), washed, and dried in a vacuum.

### Synthesis of sHMS Nanoparticles

sHMS nanoparticles were synthesized via the following method. 100 mg of solid silica nanoparticles were added into 50 mL of water via ultrasonication (1000 W, 5 min). 750 mg of CTAB (>99%) and 30 mg of NaOH were added into the dispersion and stirred at 60 °C for 1 h, then 4 mL of TEOS/BTES (3: 1, v/v) in 16 mL of cyclohexane mixture were injected into the dispersion and stirred. After stirring for a further 24 h, the disulfide‐doped mesoporous silica‐wrapped solid silica nanoparticles were collected via centrifugation (8000 rpm, 0.5 h), washed, and dried in a vacuum for 4 h. Afterward, 200 mg of disulfide‐doped mesoporous silica‐wrapped solid silica nanoparticles were dispersed in 20 mL of NaOH (0.1 m) and stirred at 60 °C for 2 h, then the products were collected by centrifugation (8000 rpm, 0.5 h), washed with ethanol/water, and dried in a vacuum for 4 h. In addition, the sphere‐like sHMS nanoparticles were prepared with the same method except that 30 mg of NaOH should be not added and 2 h for etching should be replaced with 0.5 h.

### Synthesis of AsHMS‐TA/Fe^III^@NK Nanoparticles

0.5 mL of AIPH (10 mg mL^−1^) was added to 10 mL of sHMS dispersions (2 mg mL^−1^) and stirred for 8 h. The prepared AsHMS NPs nanoparticles were collected by centrifugation (8000 rpm, 20 min) and ethanol/water to remove the unencapsulated AIPH.

The AsHMS‐TA/Fe^III^ nanogenerators were prepared by dispersing 10 mg of AsHMS into 10 mL of ethanol/water (1: 9, v/v), then adding 100 µL of TA (0.02 m) aqueous solution and 100 µL of Fe(NO)_3_.9H_2_O (0.06 m) aqueous solution under stirring. The solution pH was adjusted to ≈7.0 using 1 m NaOH under stirring. Finally, the prepared AsHMS‐TA/Fe^III^ nanoparticles were collected by centrifugation (12 000 rpm, 5 min) and washed with ethanol thrice to remove extra TA and unreacted ion.

The AsHMS‐TA/Fe^III^@NK nanogenerators were prepared by the extrusion of NK cell membranes on AsHMS‐TA/Fe^III^ nanoparticles. First, the NK cell membranes were prepared according to a well‐established procedure^[^ ^48^, ^49^ ^]^ and re‐dispersed in PBS. Subsequently, 400 µL of NK cell membranes (0.75 mg mL^−1^) was added into the 3 mL of AsHMS‐TA/Fe^III^ dispersions (0.25 mg mL^−1^). After sonication for 10 min at 4 °C and extrusion via 0.80, 0.45, and 0.20 µm filters (Nuclepore Track‐Etch Membrane Filter, USA), the dispersions were centrifuged (12 000 rpm, 10 min, 4 °C), washed with cold PBS, and re‐dispersed in 10 mL of PBS by ultrasonication at 400 W in an ice bath for 5 min. The prepared AsHMS‐TA/Fe^III^@NK nanoparticles were stored at 4 °C for further use.

### Characterization

SEM images were acquired from a SU‐70 electron microscope (Japan). TEM images, high‐angle annular dark‐field images, EDS element mappings were obtained on a Talos F200 electron microscope (US). Nitrogen adsorption–desorption isotherm and pore‐size distribution were performed by a Tristar 3000 system (US). XRD was tested on an Ultima IV X‐ray diffractometer (Japan). XPS spectra were recorded by Thermo Fisher Scientific K‐Alpha+ (US). FTIR spectra were recorded by a Nicolet Avatar 360 spectrometer (US). UV–vis–NIR absorption spectra were performed on a UV‐3600 spectrometer (Japan). Element concentration was determined by an Agilent 5100 inductively coupled plasma optical emission spectrometer (Switzerland). ESR spectra were performed on a Bruker EMX‐10/12 spectrometer (Germany). CLSM images were acquired on a Leica TCS SP5 CLSM (Germany). NIR fluorescence images were acquired by a Series II 900/1700 NIR‐II small animal imaging system (China). NIR photoacoustic images were acquired by a VisualSonics Vevo‐2100 system (Canada).

### Cell Culture

Human hepatocarcinoma cell line HepG2 cells and murine macrophage cell line RAW 264.7 cells were purchased from American Type Culture Collection and cultured in Dulbecco's modified eagle medium supplemented with 10% FBS and 1% penicillin/streptomycin at 37 °C under 5% CO_2_. For hypoxic conditions, the cells were treated into in a hypoxia incubator with 5% CO_2_ and 1% O_2_ at 37 °C.

### In Vitro Cellular Uptake

The cellular uptake assay was assessed using CLSM and flow cytometry. For CLSM observation, cells were seeded at a density of 3 × 10^4^ cells/well in a 12‐well plate and cultured for 24 h. Then cells were incubated with AsHMS‐TA/Fe^III^@NK for 1, 2, and 4 h, respectively. After incubation, the culture medium was removed and the cells were washed with PBS thrice. Cell nuclei were stained with DAPI for 10 min and washed with PBS thrice. For flow cytometry, cells were seeded in 12‐well plates and incubated with AsHMS‐TA/Fe^III^@NK for 1, 2, and 4 h. After incubation, the culture medium was removed and the cells were washed with PBS thrice, then the resulting cells were digested and analyzed using a flow cytometer.

### In Vitro Cell Viability

For the dark‐toxicity study, HepG2 cells seeded at a density of 5 × 10^4^ cells/well in a 96‐well plate. After 12 h of incubation, cells were exposed to AsHMS‐TA/Fe^III^@NK at different concentrations at 37 °C for 24 h. After 24 h of incubation, CCK‐8 assay was performed according to the manufacturer's instructions. For photo‐toxicity study, HepG2 cells were seeded at a density of 5 × 10^4^ cells/well in a 96‐well plate. After 12 h of incubation, cells were exposed to AsHMS‐TA/Fe^III^@NK with different concentrations at 37 °C for 12 h. Then, 100 µL of the original medium was replaced with the equivalent volume of fresh medium, and the cells were exposed to 808 nm laser irradiation (0.5 W cm^−2^, 5 min). After 12 h of further incubation, the CCK‐8 assay was performed according to the manufacturer's instructions.

### Apoptosis Analysis

HepG2 cells were seeded at a density of 3.0 × 10^4^ cells per well in a 12‐well plate. The next day, the cells were treated with AsHMS‐TA/Fe^III^@NK. After 12 h of incubation, HepG2 cells were rinsed with PBS buffer and irradiated with 808 nm laser (0.5 W cm^−2^) for 5 min. After incubation for another 12 h, the HepG2 cells were digested with trypsin, washed, resuspended in binding buffer, and co‐incubated with Annexin V‐FITC/PI according to the manufacturer's instructions. Then cells were rinsed with PBS and then analyzed using a flow cytometer. Lastly, the harvested data was analyzed using FlowJo software.

### Animal Model

BALB/c female nude mice were supplied by the Experimental Animal Center of Fujian Medical University. Animal procedures were conducted according to the “National animal management regulations of China” and approved by the Animal Ethics Committee of Mengchao Hepatobiliary Hospital of Fujian Medical University. HepG2 tumor‐bearing nude mice were constructed by subcutaneous injection of HepG2 cells into the right thigh of mice until the tumor size reached ≈100 mm^3^.

### In Vivo Multimodal Imaging

HepG2 tumor‐bearing BALB/c nude mice were intravenously administrated with the AsHMS‐TA/Fe^III^@NK when the tumor size reached ≈100 mm^3^. For in vivo NIR fluorescence imaging, the mice were imaged by a NIR‐OPTICS Series III 900/1700 at predesigned time points. At 24 h post‐injection, the major organs (heart, liver, spleen, lung, and kidney) and tumors were harvested for ex vivo fluorescence imaging. For in vivo NIR photoacoustic imaging, the tumor areas of mice were imaged by a VisualSonics Vevo‐2100 system at predesigned time points. For in vivo infrared photothermal imaging, the NIR laser (808 nm, 0.5 W cm^−2^, 5 min) was irradiated at the tumor sites at 12 h, and the mice were imaged by a FLIR thermal imager. The tumor temperature change was also recorded.

### In Vivo Therapy

The HepG2 tumor‐bearing mice were randomly divided into six different groups (*n* = 10). The mice were intravenously injected with 0.9%NaCl, sHMS‐TA/Fe^III^, AsHMS‐TA/Fe^III^, and AsHMS‐TA/Fe^III^@NK at the equivalent concentration of sHMS‐TA/Fe^III^. At 12 h post‐injection, the mice were irradiated using 808 nm laser at 0.5 W cm^−2^ for 5 min. The maximum and minimum diameters of tumors of each group were measured using a vernier caliper. The tumor volume was calculated using the equation: *V* = 1/2 × *a* × *b* ^2^, where *a* and *b* represented the maximum length and the minimum width of tumors, respectively. The body weight change and survival time were also recorded.

After observation for 21 days, the HepG2 tumor‐bearing mice were euthanized, the major organs and tumors were dissected to perform H&E staining. Additionally, the liver and renal function of mice after different treatments were further verified by detecting the level of alanine transaminase (ALT), aspartate aminotransferase (AST), blood urea nitrogen (BUN), and creatinine (CREA).

### Statistical Analysis

Data were obtained from at least three independent measurements (*n* ≥ 3). Data were shown as mean ± standard error (SD) of triplicates unless otherwise indicated. Statistical analysis was performed using a two‐sided Student's *t*‐test. *p* < 0.05, *p* < 0.01, and *p* < 0.001 were considered to be statistically significant with noting by *, **, and ***, respectively.

## Conflict of Interest

The authors declare no conflict of interest.

## Supporting information

Supporting Information

## Data Availability

Research data are not shared.
