# Supplementary material for: Natural Killer Cell Membrane‐Cloaked Virus‐Mimicking Nanogenerator with NIR‐Triggered Shape Reversal and •C/•OH Storm for Synergistic Thermodynamic–Chemodynamic Therapy
Source: Adv Sci (Weinh). 2021 Dec 19;9(5):2103498. doi: 10.1002/advs.202103498 (PMC8844581; doi:10.1002/advs.202103498)
Supplement: Supplementary file 1 — Supporting Information [file ADVS-9-2103498-s001.pdf]

ADVANCED SCIENCE
Open Access

Supporting Information

for *Adv. Sci.*, DOI: 10.1002/advs.202103498

Natural Killer Cell Membrane-Cloaked Virus-Mimicking Nanogenerator with NIR-Triggered Shape Reversal and •C/•OH Storm for Synergistic Thermodynamic-Chemodynamic Therapy

Jinyan Lin, Yang Li, Peiyuan Wang, Ming Wu, Fukai Zhu, Yun Zhang, Zhenqing Hou, Jingfeng Liu, Xiaolong Liu**

Supporting Information

Natural Killer Cell Membrane-Cloaked Virus-Mimicking Nanogenerator with NIR-Triggered Shape Reversal and •C/•OH Storm for Synergistic Thermodynamic-Chemodynamic Therapy

Jinyan Lin,[#] Yang Li,[#*] Peiyuan Wang, Ming Wu, Fukai Zhu, Yun Zhang, Zhenqing Hou, Jingfeng Liu, Xiaolong Liu[*]

Dr. J. Lin, Prof. Y. Li, Prof. P. Wang, Prof. M. Wu, Prof. J. Liu, Prof. X. Liu

The United Innovation of Mengchao Hepatobiliary Technology Key Laboratory of Fujian Province, Mengchao Hepatobiliary Hospital of Fujian Medical University, Fuzhou 350025, P. R. China;

E-mail: xiaoloong.liu@gmail.com

Prof. Y. Li, Prof. P. Wang, Prof. Y. Zhang, Prof. J. Liu, Prof. X. Liu

CAS Key Laboratory of Design and Assembly of Functional Nanostructures, Fujian Institute of Research on the Structure of Matter, Chinese Academy of Sciences, Fuzhou 350002, P.R. China

E-mail: li.yang@fjirsm.ac.cn

Prof. J. Liu

Fujian Cancer Hospital and Fujian Medical University Cancer Hospital, Fuzhou 350014, P.R. China

Prof. Y. Li, Prof. P. Wang, Prof. Y. Zhang, Prof. J. Liu, Prof. X. Liu

Department of Translational Medicine, Xiamen Institute of Rare Earth Materials, Chinese Academy of Sciences, Xiamen 361024, P. R. China.

F. Zhu, Prof. Z. Hou

College of Materials, Xiamen University, Xiamen 361005, P. R. China.

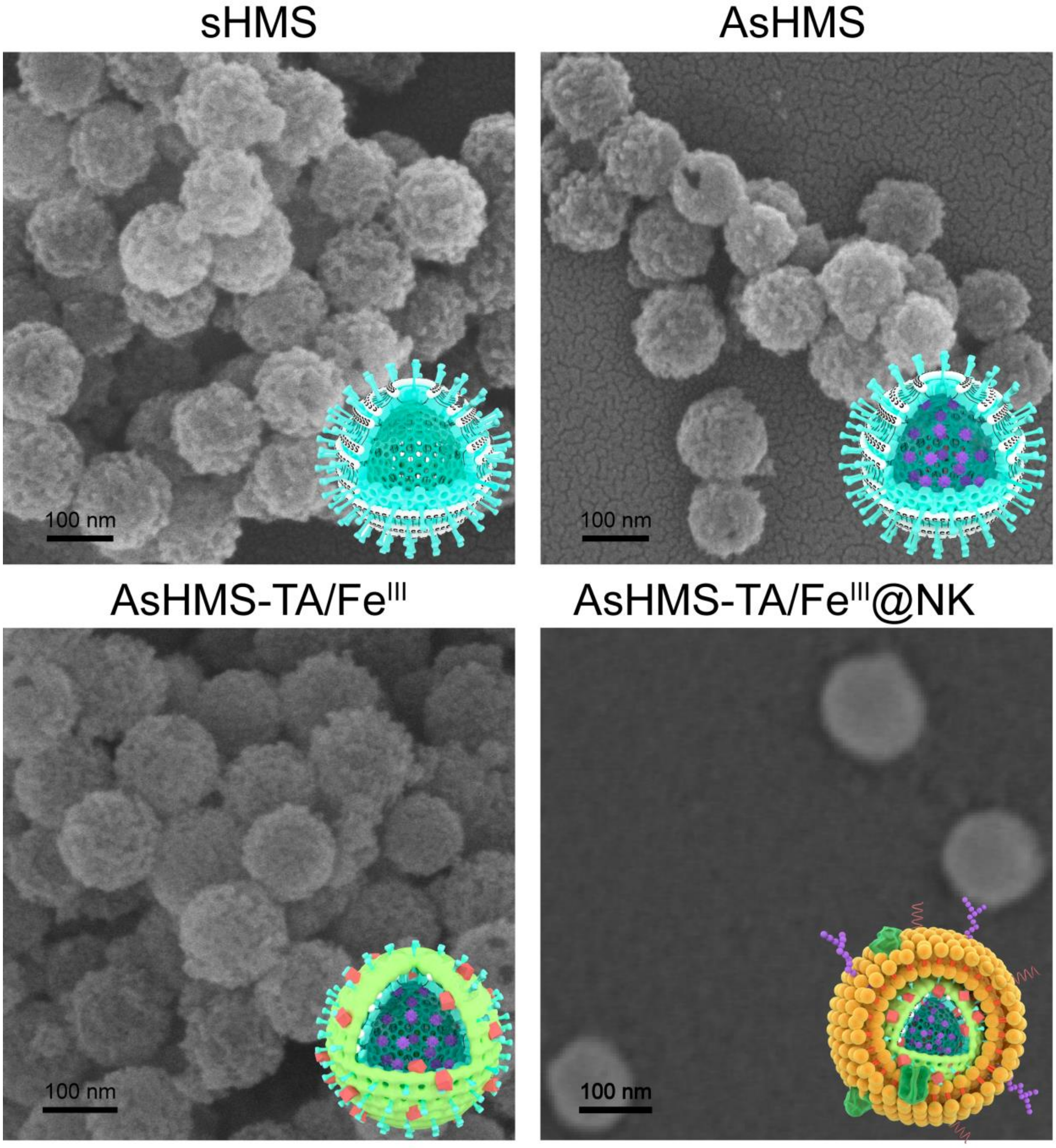

Figure S1. SEM images of sHMS, AsHMS, AsHMS-TA/Fe^{III}, and AsHMS-TA/Fe^{III}@NK.

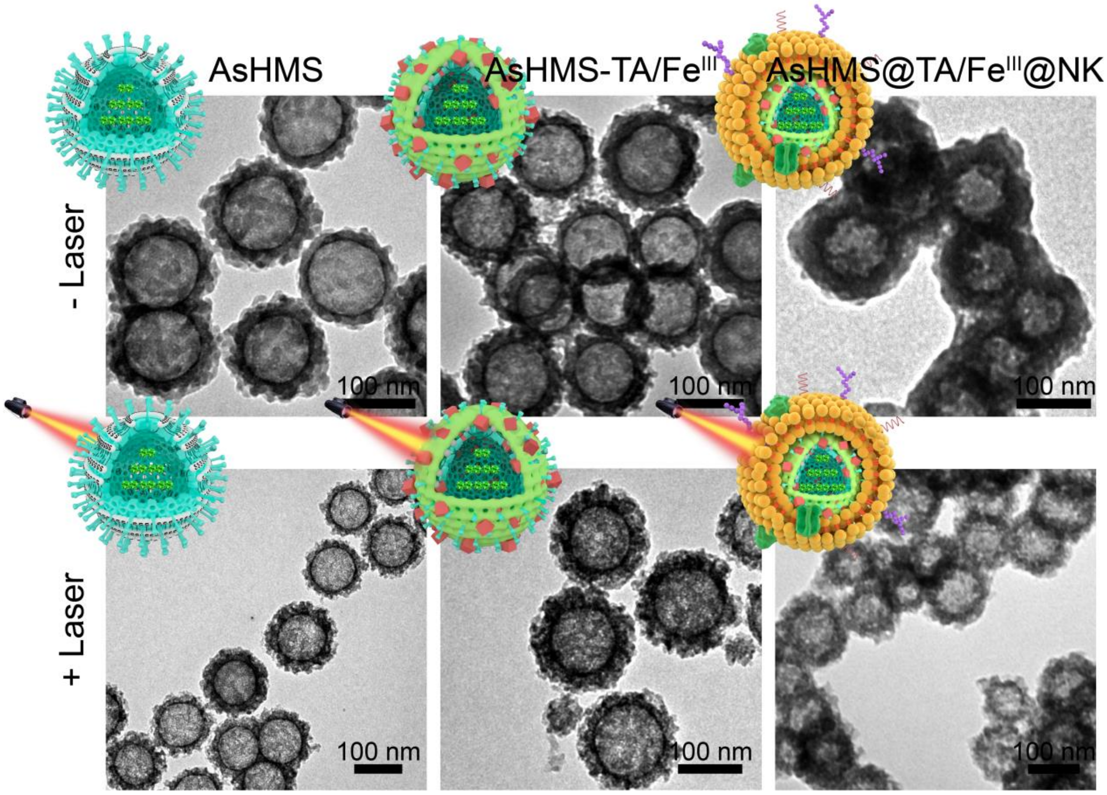

Figure S2. TEM images of AsHMS, AsHMS-TA/Fe^{III}, and AsHMS-TA/Fe^{III}@NK with/without NIR laser irradiation (TEM images without NIR laser irradiation are derived from Figure 1A).

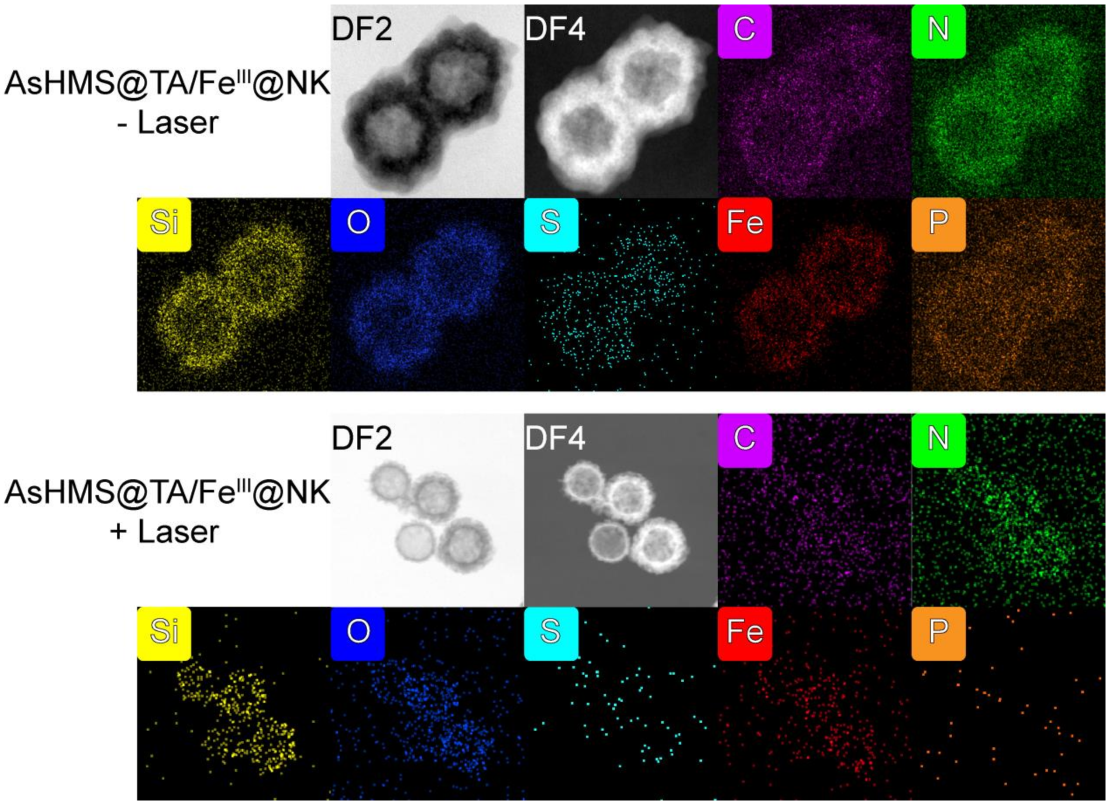

Figure S3. EDS element mappings of AsHMS-TA/Fe^{III}@NK with/without NIR laser irradiation (EDS element mappings without NIR laser irradiation are derived from Figure 1B). The AsHMS-TA/Fe^{III}@NK was exposed to NIR laser irradiation (808 nm, 0.5 W·cm^{-2}, 5 min) and then washed by centrifugation (8000 rpm, 20ºC, 10 min). Compared with the AsHMS-TA/Fe^{III}@NK without NIR laser irradiation, the P element was disappeared in AsHMS-TA/Fe^{III}@NK with NIR laser irradiation whereas the Fe element was still preserved. This result indicated the re-exposure of TA/Fe^{III} layer on the surface after NIR laser irradiation.

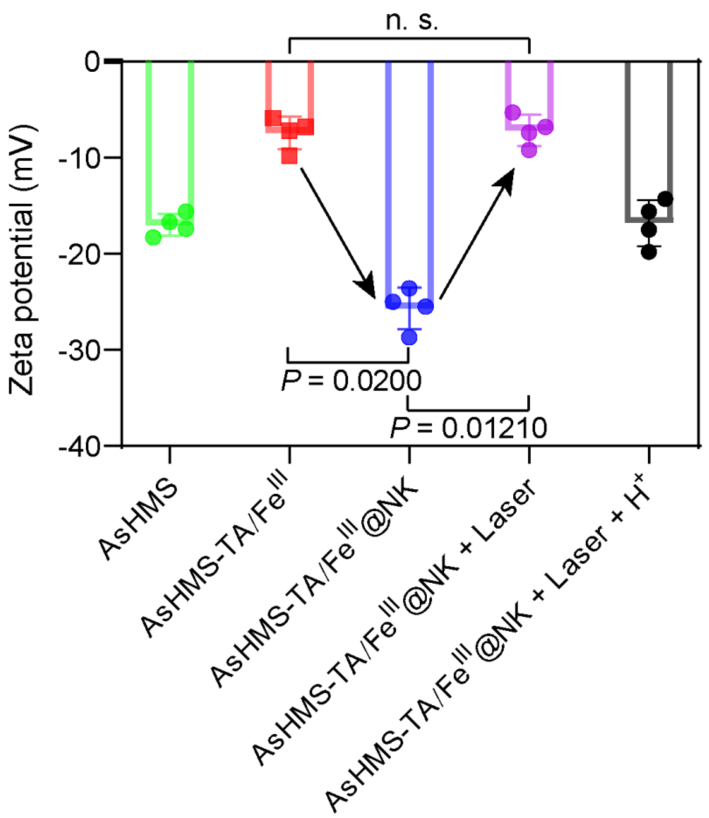

Figure S4. Zeta potential change of AsHMS, AsHMS-TA/Fe^{III}, AsHMS-TA/Fe^{III}@NK with/without external stimuli treatment. For AsHMS-TA/Fe^{III}@NK with laser irradiation, the AsHMS-TA/Fe^{III}@NK was exposed to NIR laser irradiation (808 nm, 0.5 W·cm^{-2}, 5 min) and then washed by centrifugation (8000 rpm, 20ºC, 10 min). For AsHMS-TA/Fe^{III}@NK with laser irradiation/acidity treatment, the AsHMS-TA/Fe^{III}@NK was exposed to NIR laser irradiation (808 nm, 0.5 W·cm^{-2}, 5 min), washed by centrifugation (8000 rpm, 20ºC, 10 min), exposed to acidic treatment (pH 5.0, 1 h), and then washed again by centrifugation (8000 rpm, 20ºC, 10 min).

When the AsHMS-TA/Fe^{III}@NK was exposed to NIR laser irradiation and washed by centrifugation, the surface charge was decreased from ~-25.7 mV to ~-7.2 mV, which was very similar to that of AsHMS-TA/Fe^{III} (~7.4 mV) rather than AsHMS (~17.0 mV). These results indicated the re-exposure of TA/Fe^{III} layer on the surface after NIR laser irradiation.

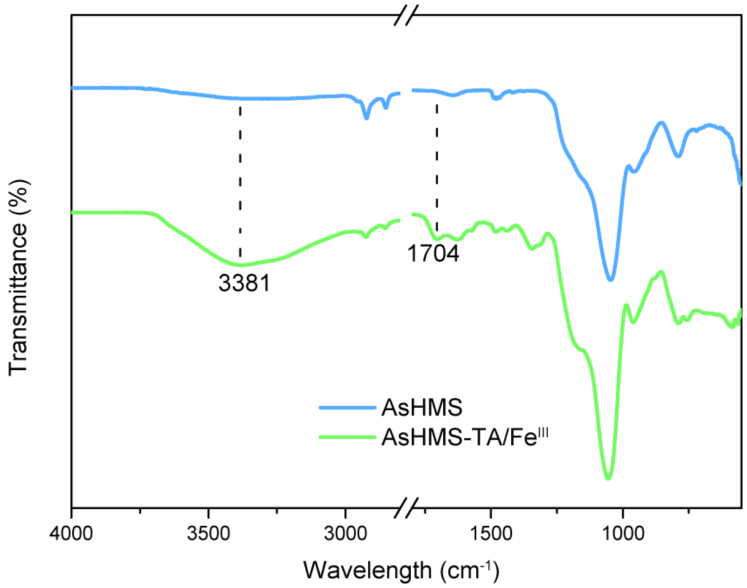

Figure S5. FT-IR spectra of AsHMS and AsHMS-TA/Fe^{III}.

Compared with the absorption peak in the spectrum of AsHMS, two new absorption peaks appeared at 3381 cm^{-1} and 1704 cm^{-1} in the spectrum of AsHMS-TA/Fe^{III}, which was respectively assigned to the O-H stretching vibration of phenolic hydroxyl group and C=O stretching vibration of carbonyl group due to the introduction of TA.

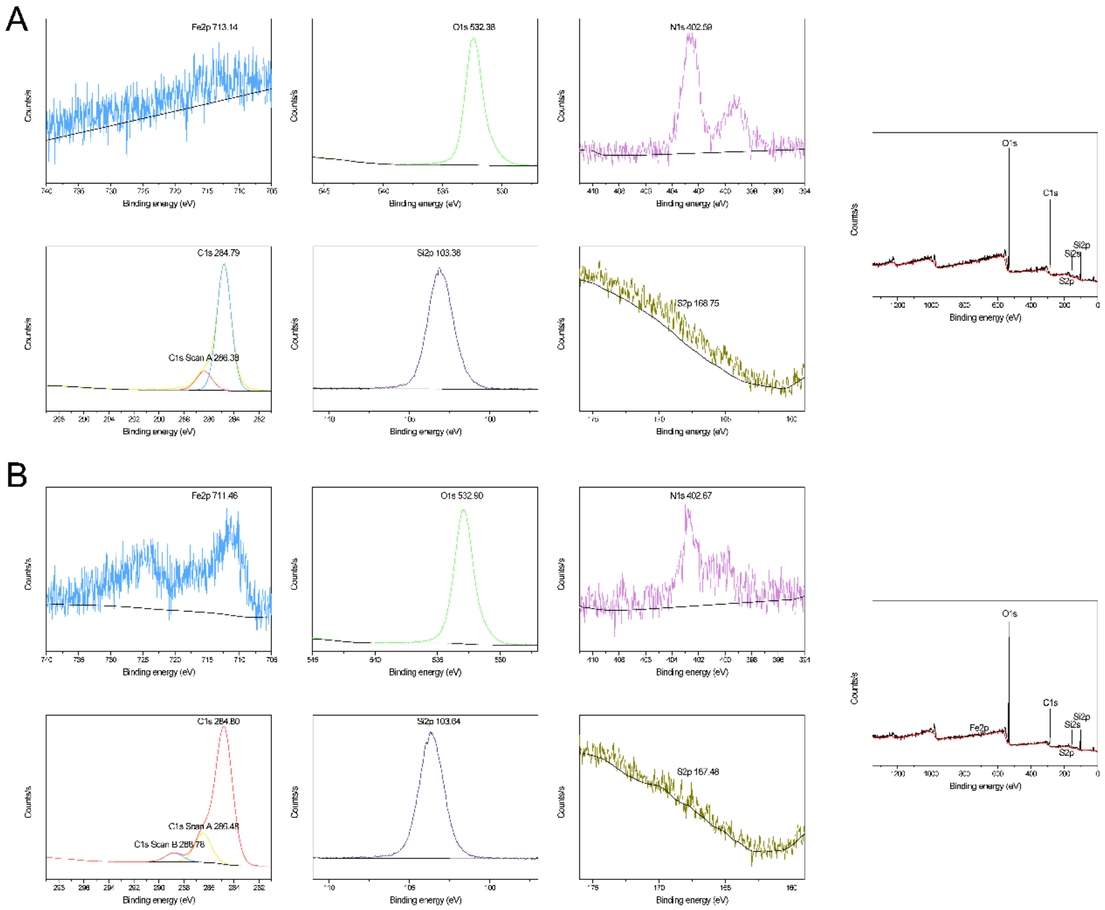

Figure S6. XPS spectra of (A) AsHMS and (B) AsHMS-TA/Fe^{III}.

Compared with AsHMS, the XPS spectrum of AsHMS-TA/Fe^{III} indicated the presence of Fe element.

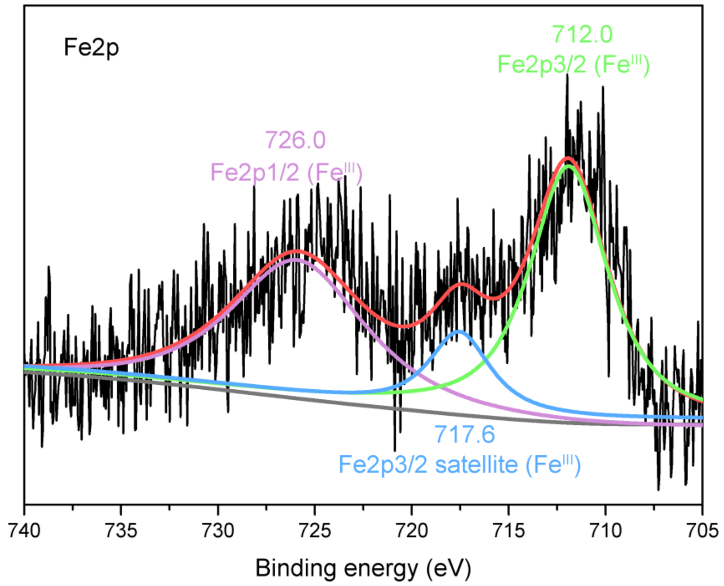

Figure S7. XPS high-resolution spectra of Fe2p region of AsHMS-TA/Fe^{III}.

The Fe2p spectrum of AsHMS-TA/Fe^{III} exhibited two peaks centered at 712.0 and 726.0 eV, which were assigned to Fe2p3/2 (Fe^{III}) and Fe2p1/2 (Fe^{III}), respectively. The result proved the Fe^{3+} valence state of AsHMS-TA/Fe^{III}.

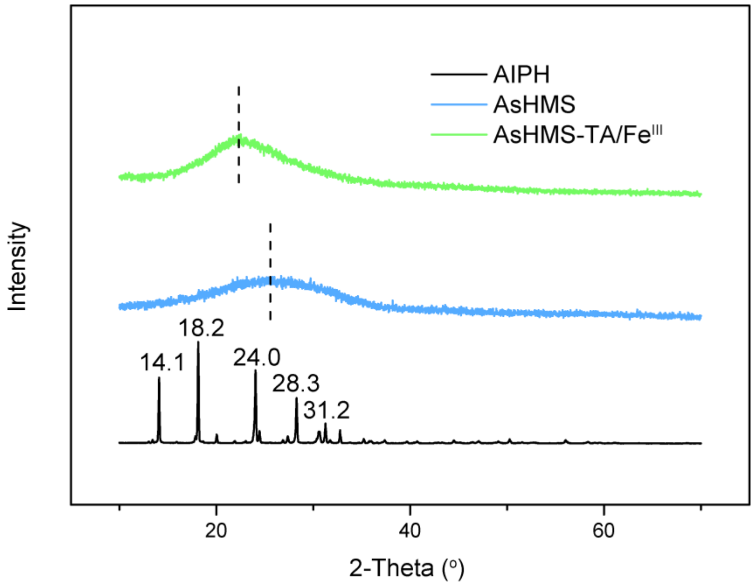

Figure S8. XRD spectra of AIPH, AsHMS, and AsHMS-TA/Fe^{III}.

The microstructures of AIPH, AsHMS, and AsHMS-TA/Fe^{III} were analyzed by XRD spectra. The sharp crystalline peaks were observed in the XRD spectrum of AIPH. By contrast, the crystalline signals for AIPH clearly disappeared in the spectrum of AsHMS and AsHMS-TA/Fe^{III}, indicating that the strong crystallization was significantly restricted in sHMS and sHMS-TA/Fe^{III}. The result demonstrated that AIPH was molecularly dispersed within the sHMS and sHMS-TA/Fe^{III}.

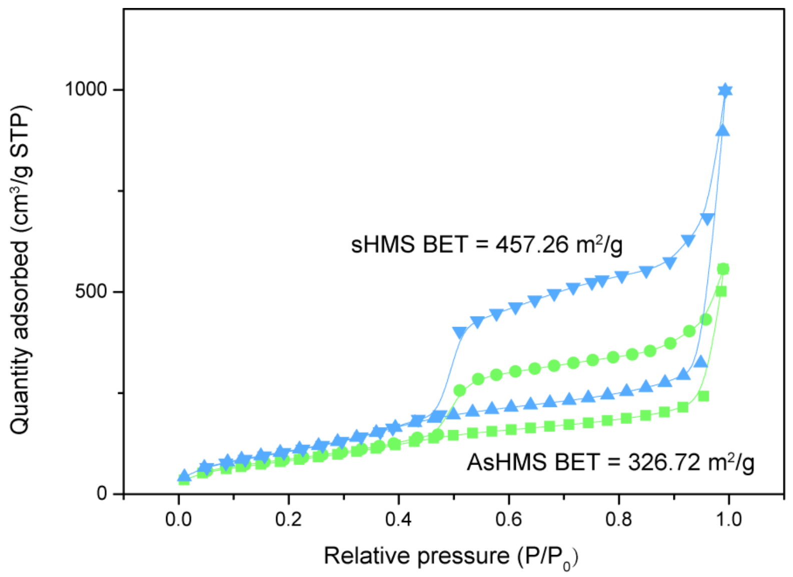

Figure S9. N_2 adsorption-desorption isotherms of sHMS and AsHMS.

Compared with the sHMS, the BET surface area was significantly decreased in AsHMS, indicating the successful loading of AIPH within the sHMS nanoparticles.

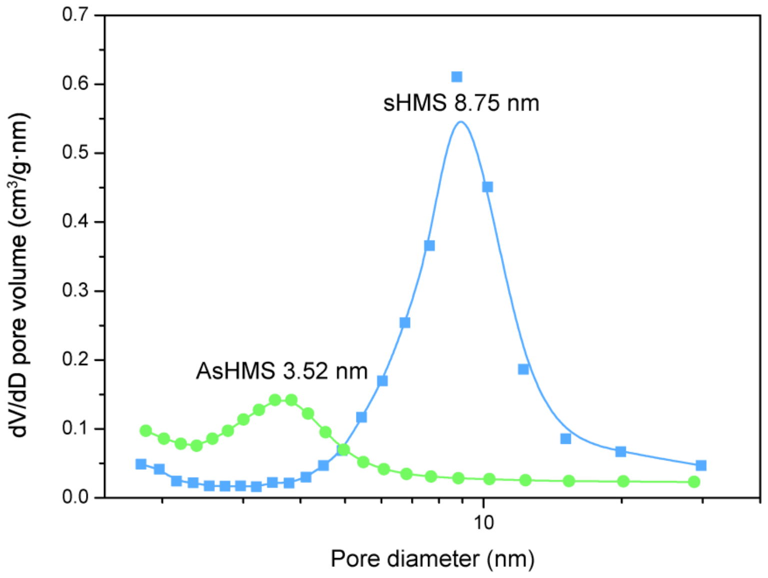

Figure S10. Pore diameter distributions of sHMS and AsHMS.

As comparison with the sHMS nanoparticles, the pore diameter of AsHMS nanoparticles was obviously declined, demonstrating that the AIPH molecules were successfully loaded into the pore channels of sHMS nanoparticles.

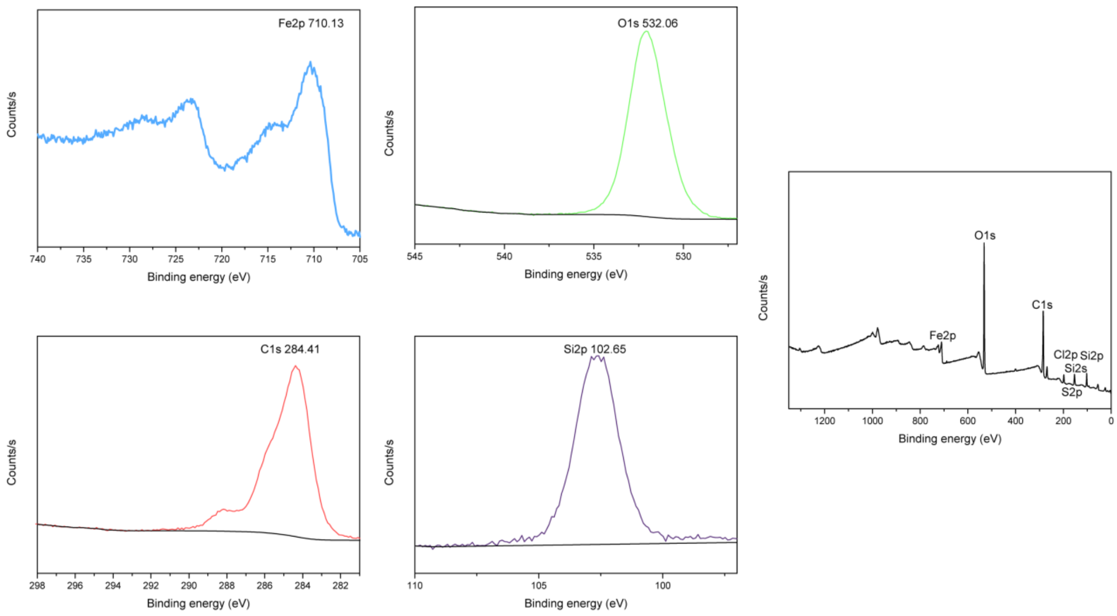

Figure S11. XPS spectra of AsHMS-TA/Fe^{III} after acidic treatment (pH 5.0).

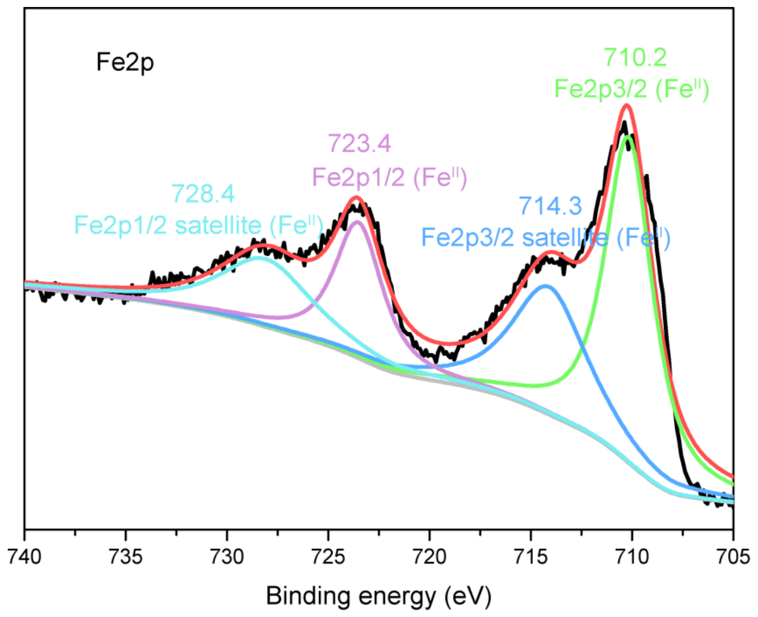

Figure S12. XPS high-resolution spectra of Fe2p region of AsHMS-TA/Fe^{III} after acidic treatment (pH 5.0).

The Fe2p spectrum of AsHMS-TA/Fe^{III} after acidic treatment (pH 5.0) exhibited two peaks centered at 710.2 and 723.4 eV, which were assigned to Fe2p3/2 (Fe^{II}) and Fe2p1/2 (Fe^{II}), respectively. The result indicated the conversion of Fe^{3+} into Fe^{2+} by TA (acidity-activated reducing agent) under acidic condition.

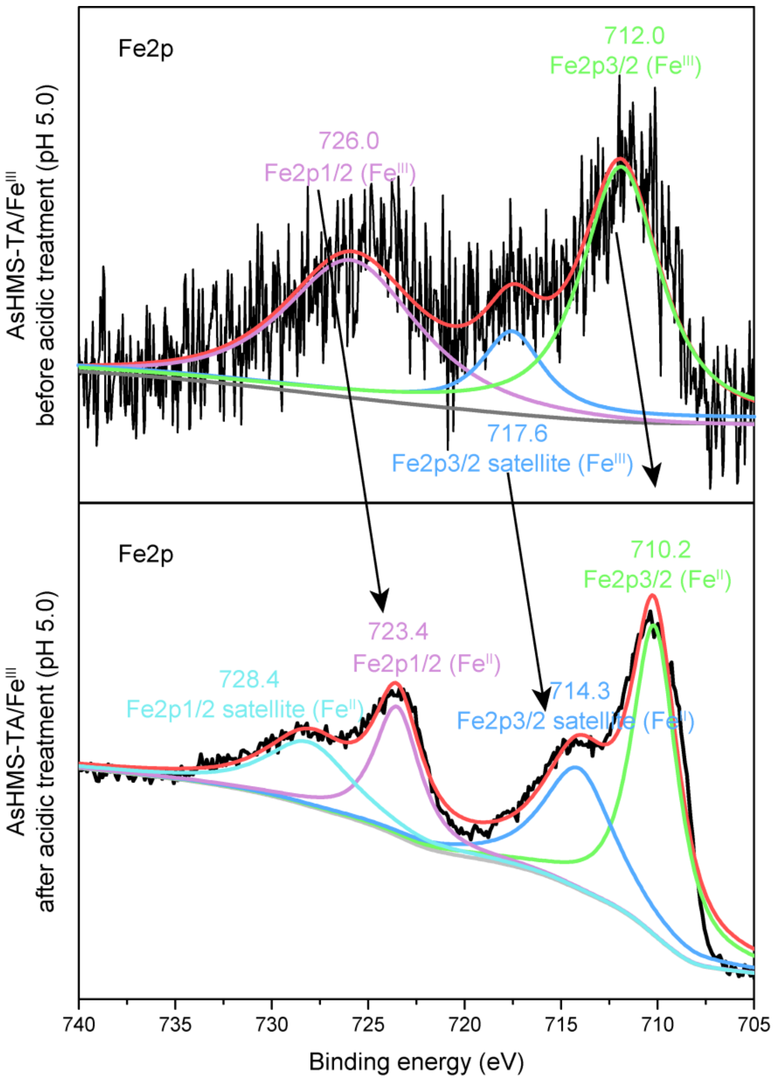

Figure S13. XPS high-resolution spectra of Fe2p region of AsHMS-TA/Fe^{III} before and after acidic treatment (pH 5.0).

By comparison with the AsHMS-TA/Fe^{III}, the binding energy of AsHMS-TA/Fe^{III} after acidic treatment (pH 5.0) in XPS high-resolution spectra of Fe2p region shifted to the lower binding energy side. The result proved the conversion of Fe^{3+} into Fe^{2+} by TA under acidic condition.

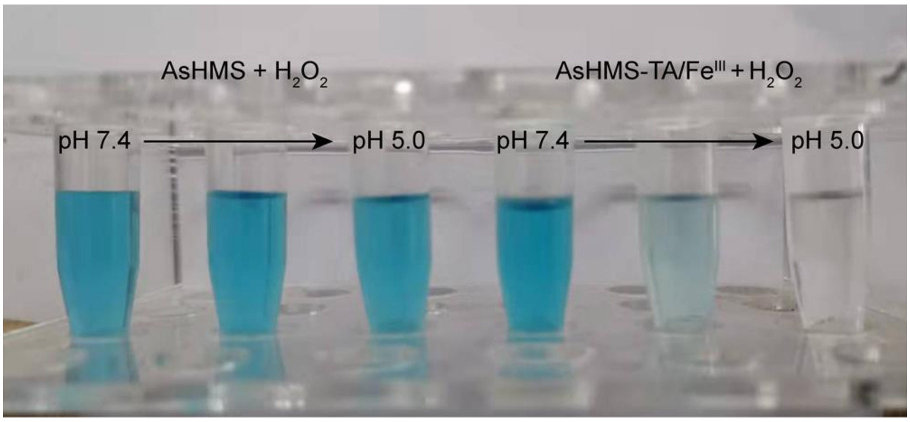

Figure S14. Photographs of methylene blue (MB) treated with AsHMS-TA/Fe^{III} + H_2O_2 at pH 7.4, 6.0, and 5.0. MB treated with AsHMS + H_2O_2 at pH 7.4, 6.0, and 5.0 was used as controls.

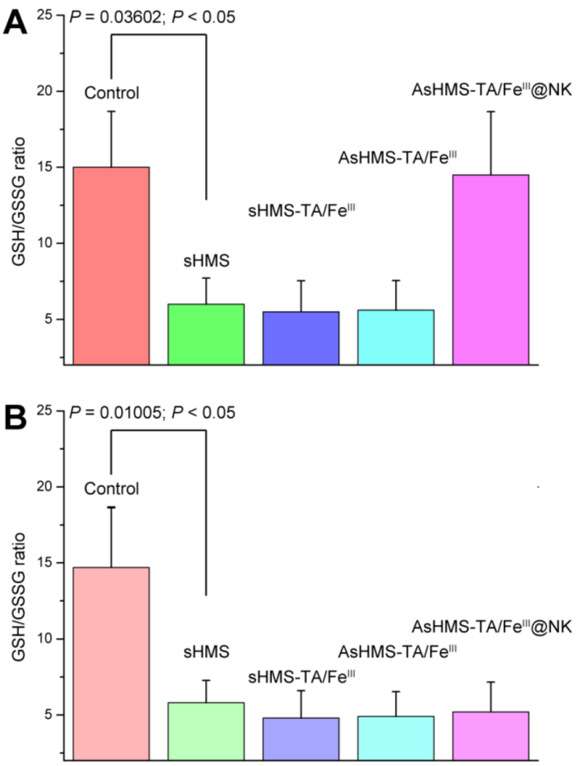

Figure S15. GSH/GSSG ratio in HepG2 cells incubated with PBS, sHMS, sHMS, AsHMS-TA/Fe^{III}, and AsHMS-TA/Fe^{III}@NK with/without laser irradiation for 4 h (n = 4).

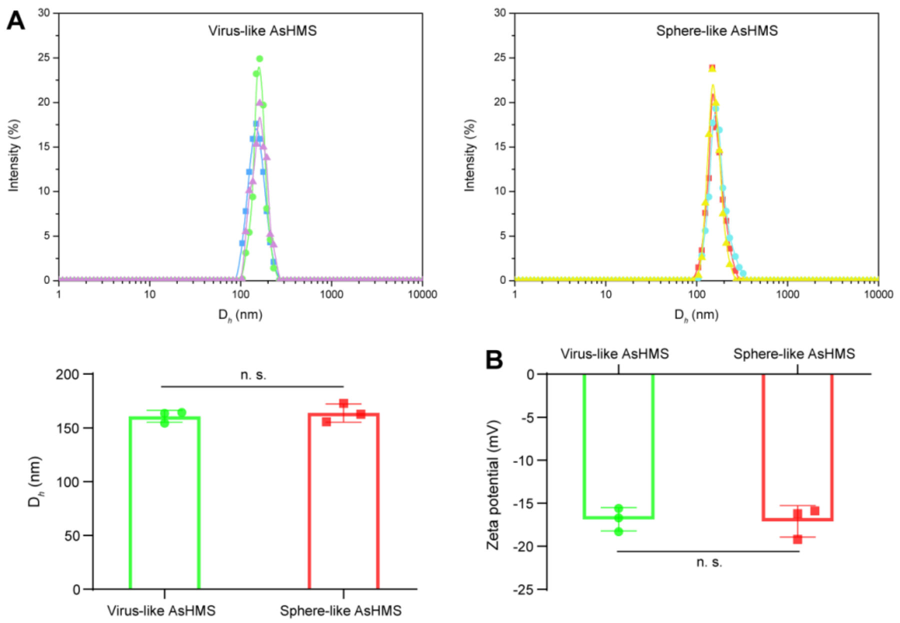

Figure S16. (A) D_h distribution and average D_h of virus-like and sphere-like AsHMS. (B) Zeta potential of virus-like and sphere-like AsHMS (n = 3).

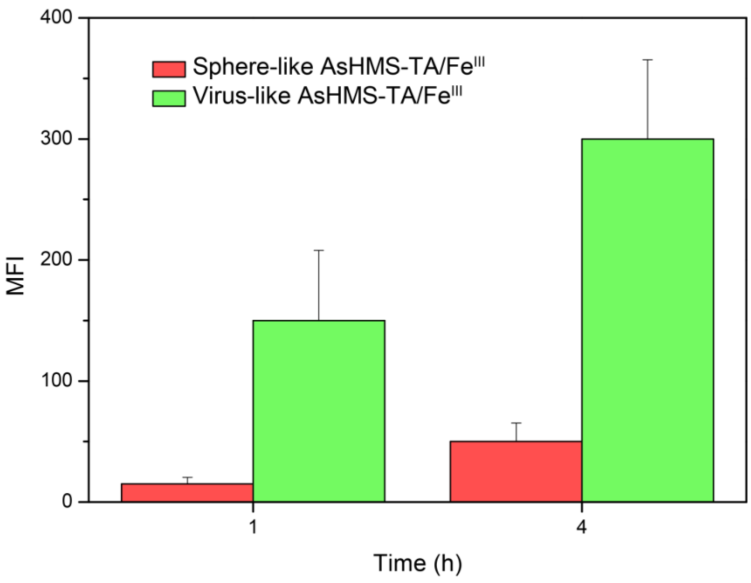

Figure S17. Mean fluorescence intensity (MFI) of HepG2 cells incubated with sphere-like and virus-like nanogenerators for 1 and 4 h.

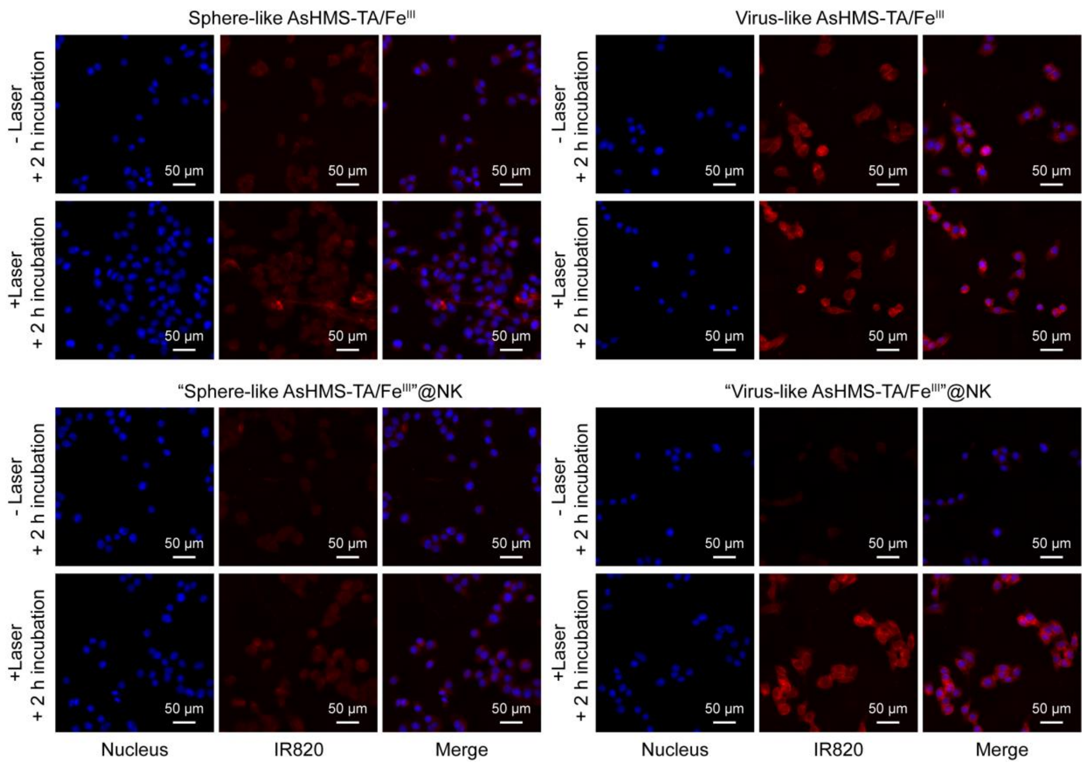

Figure S18. CLSM images of HepG2 cells incubated with "sphere-like AsHMS-TA/Fe^{III}"@NK, "virus-like AsHMS-TA/Fe^{III}"@NK, sphere-like AsHMS-TA/Fe^{III}, and virus-like AsHMS-TA/Fe^{III} for 2 h with/without NIR laser irradiation pretreatment (808 nm, 0.5 W·cm^{-2}, 5 min).

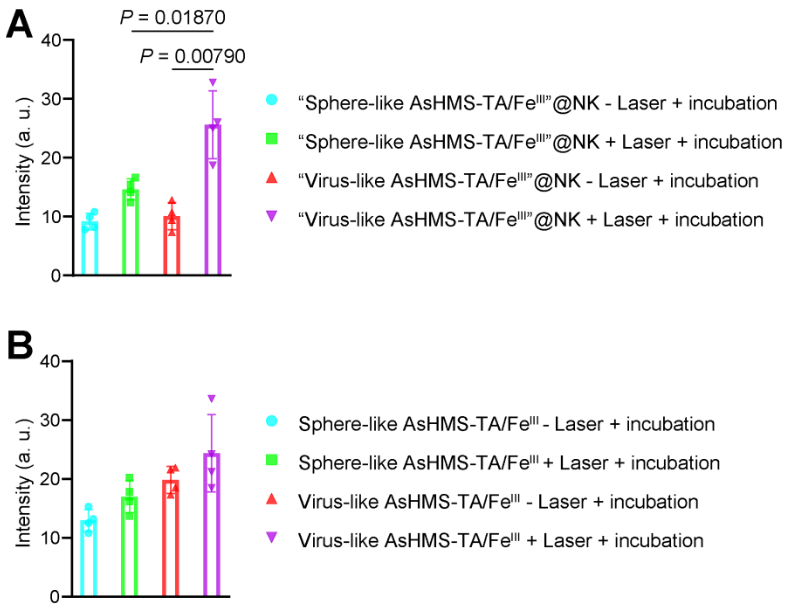

Figure S19. Mean fluorescence intensity of HepG2 cells incubated with "sphere-like AsHMS-TA/Fe^{III}"@NK, "virus-like AsHMS-TA/Fe^{III}"@NK, sphere-like AsHMS-

TA/Fe^{III}, and virus-like AsHMS-TA/Fe^{III} for 2 h with/without NIR laser irradiation pretreatment (808 nm, 0.5 W·cm^{-2}, 5 min). The fluorescence intensity was analyzed by Image J.

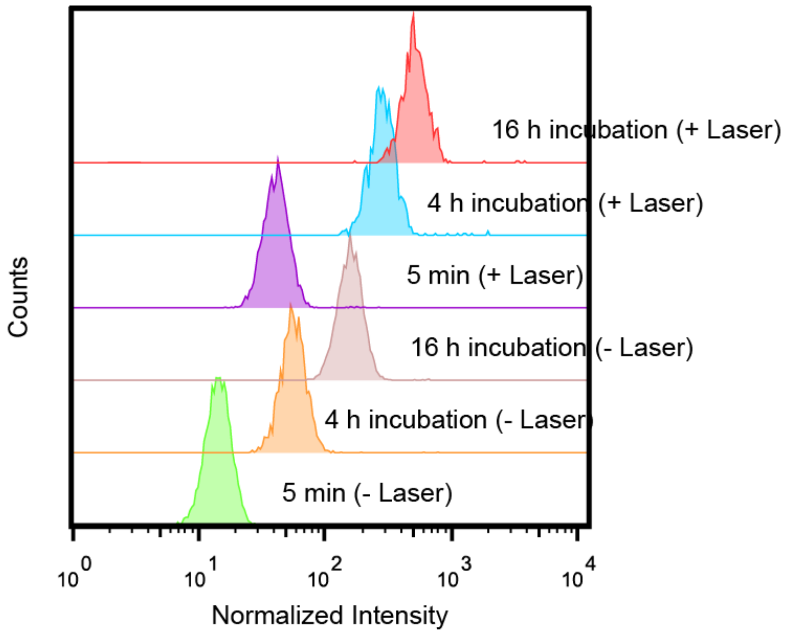

Figure S20. Flow cytometry profiles of HepG2 cells incubated with sphere-like AsHMS-TA/Fe^{III}@NK nanogenerators for different incubation time periods with/ without NIR laser irradiation.

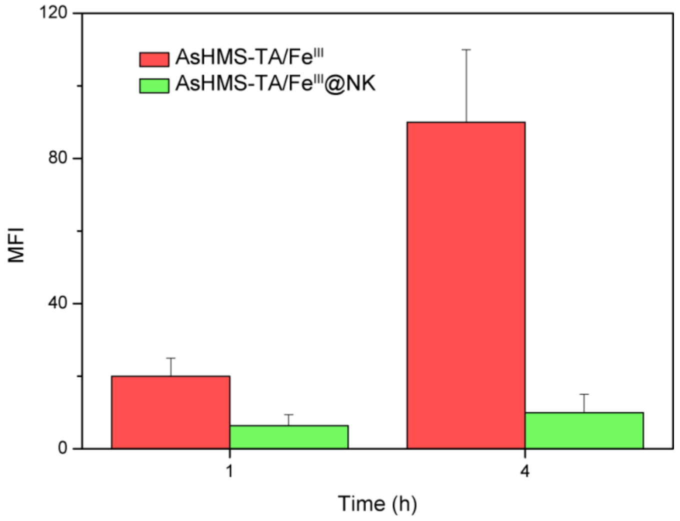

Figure S21. Mean fluorescence intensity (MFI) of macrophage RAW 264.7 cells incubated with virus-like AsHMS-TA/Fe^{III} and sphere-like AsHMS-TA/Fe^{III}@NK for

1 and 4 h.

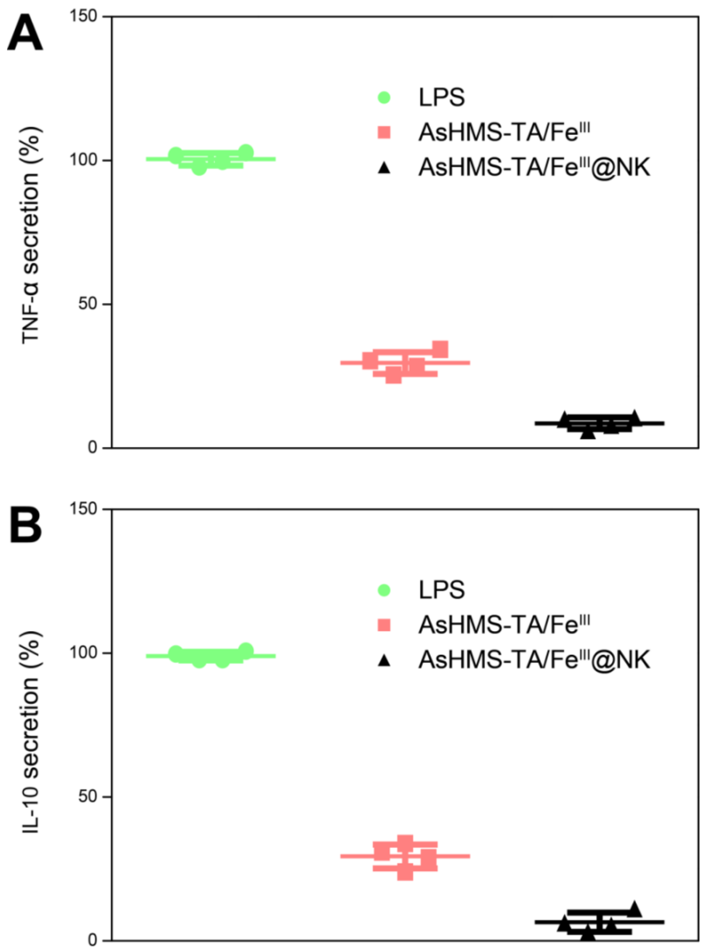

Figure S22. Quantification of (A) TNF-α and (B) IL-10 secretion by macrophages.

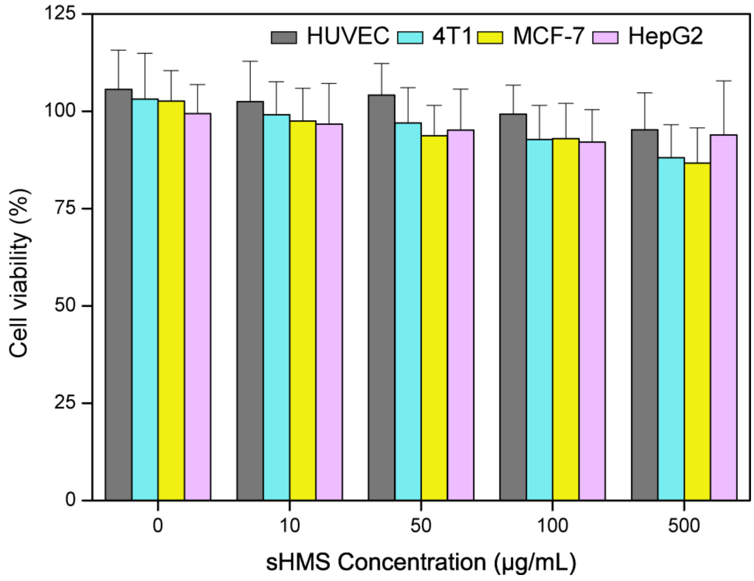

Figure S23. Viability of HUVEC, 4T1, MCF-7, and HepG2 cells treated with AsHMS-TA/Fe^{III}@NK at different sHMS concentrations for 24 h.

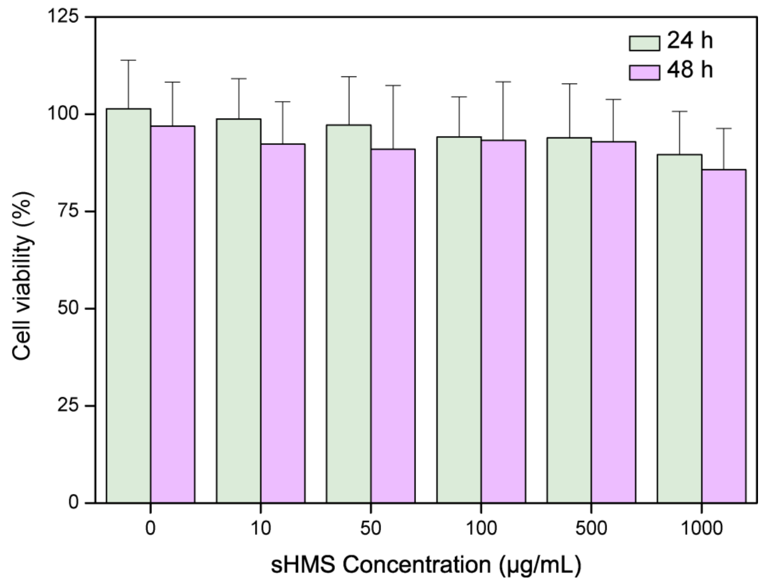

Figure S24. Viability of HepG2 cells treated with AsHMS-TA/Fe^{III}@NK at different sHMS concentrations for 24 and 48 h.

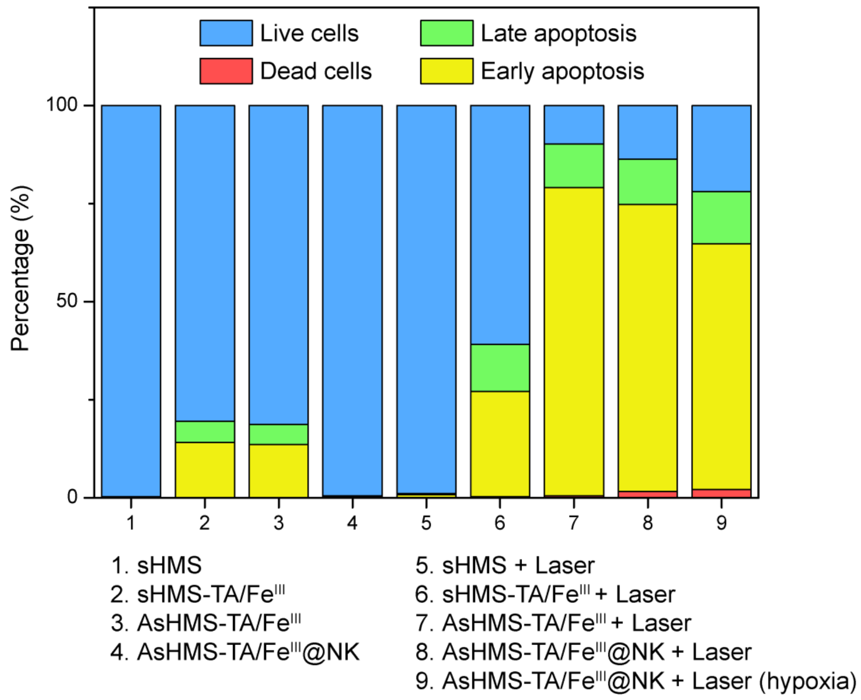

Figure S25. Percentage of cell apoptosis assays for HepG2 cells treated with sHMS, sHMS-TA/Fe^{III}, AsHMS-TA/Fe^{III}, AsHMS-TA/Fe^{III}@NK, and AsHMS-TA/Fe^{III}@NK under hypoxic condition with/without NIR laser irradiation.

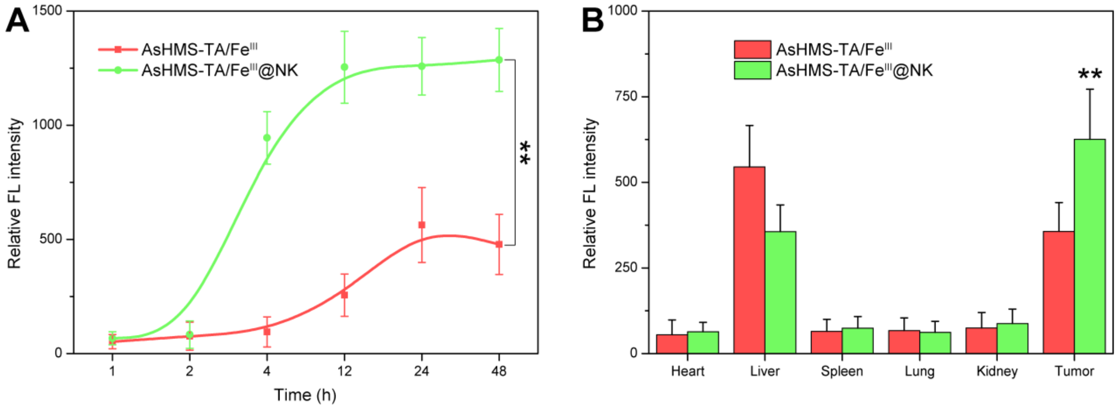

Figure S26. (A) Time-lapsed NIR fluorescence intensity in the tumor areas of HepG2 tumor-bearing nude mice after intravenous injection of AsHMS-TA/Fe^{III} and AsHMS-TA/Fe^{III}@NK. (B) NIR fluorescence intensity of excised normal and tumor tissues at 48 h post-injection. $^{**}p < 0.01$.

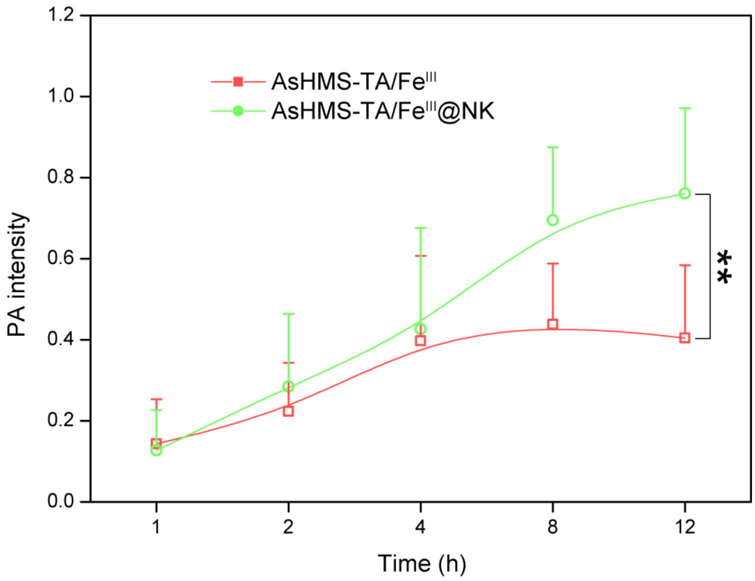

Figure S27. Time-lapsed photoacoustic intensity in the tumor areas of HepG2 tumor-bearing nude mice after intravenous injection of AsHMS-TA/Fe^{III} and AsHMS-TA/Fe^{III}@NK. $^{**}p < 0.01$.

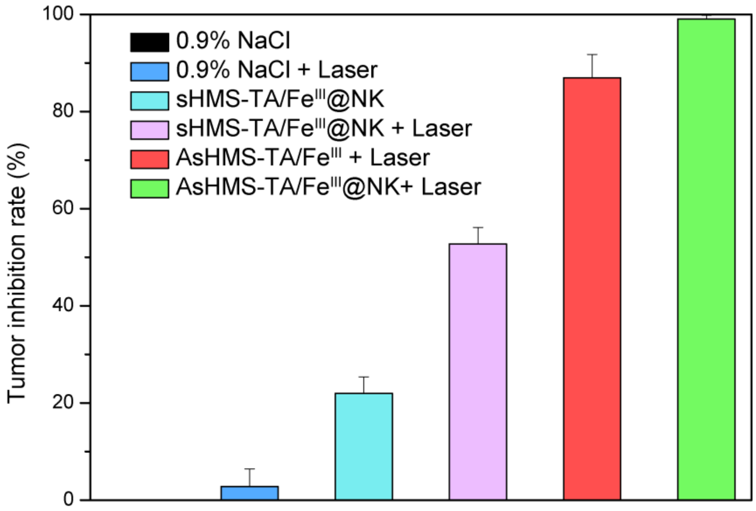

Figure S28. Tumor inhibition rate of HepG2 tumor-bearing nude mice after intravenous injection of 0.9% NaCl, sHMS-TA/Fe^{III}@NK, AsHMS-TA/Fe^{III}, and AsHMS-TA/Fe^{III}@NK with/without NIR laser irradiation.
